# Prognostic implications of tumor-infiltrating lymphocytes in non-small cell lung cancer: a systematic review and meta-analysis

**DOI:** 10.3389/fimmu.2024.1476365

**Published:** 2024-09-20

**Authors:** Qin Yan, Shuai Li, Lang He, Nianyong Chen

**Affiliations:** ^1^ Department of Head and Neck Oncology, Cancer Center and State Key Laboratory of Biotherapy, West China Hospital, Sichuan University, Chengdu, China; ^2^ Department of Radiation Oncology, Cancer Center and State Key Laboratory of Biotherapy, West China Hospital, Sichuan University, Chengdu, China; ^3^ Cancer Prevention and Treatment Institute of Chengdu, Department of Oncology, Chengdu Fifth People’s Hospital (The Second Clinical Medical College, Affiliated Fifth People’s Hospital of Chengdu University of Traditional Chinese Medicine), Chengdu, China; ^4^ School of Clinical Medicine, Chengdu University of Traditional Chinese Medicine, Chengdu, China

**Keywords:** tumor-infiltrating lymphocytes, non-small cell lung cancer, prognostic implication, systematic review, meta-analysis

## Abstract

**Background:**

Tumor-infiltrating lymphocytes (TILs) have demonstrated potential as prognostic biomarkers across various cancer types. However, their prognostic implications in non-small cell lung cancer (NSCLC) remain ambiguous.

**Methods:**

An exhaustive electronic search was executed across the Pubmed, EMBASE, Web of Science, and Cochrane Library databases to locate relevant studies published up until December 19, 2023. Studies were eligible if they assessed the association between TILs and overall survival (OS) and disease-free survival (DFS) in NSCLC patients. The OS and DFS were subsequently extracted for analysis. The prognostic significance of TILs was evaluated by calculating the Pooled Hazard Ratios (HRs) and their corresponding 95% Confidence Intervals (CIs).

**Results:**

The meta-analysis incorporated 60 studies, which collectively included 15829 NSCLC patients. The collective analysis indicated that NSCLC patients exhibiting TILs infiltration demonstrated a significantly improved OS(HR: 0.67; 95%CI: 0.55-0.81). Subgroup analyses, based on TIL subtypes (CD8+, CD3+ and CD4+), consistently revealed a favorable prognostic impact on OS. However, it was observed that FOXP3+ was correlated with a poor OS (HR: 1.35; 95% CI: 0.87-2.11).

**Conclusion:**

This comprehensive systematic review and meta-analysis substantiate the prognostic significance of TILs in patients diagnosed with NSCLC. Notably, elevated TILs infiltration correlates with a favorable prognosis, particularly among CD8+, CD3+ and CD4+ subtypes.

**Systematic review registration:**

https://www.crd.york.ac.uk/prospero/display_record.php?ID=CRD42023468089 PROSPERO, identifier CRD42023468089.

## 1 Introduction

Lung cancer stands as the most prevalent malignant neoplasm on a global scale (1), with non-small cell lung cancer (NSCLC) making up about 85% of all cases (2). The use of low-dose spiral CT has been instrumental in the early detection of lung cancer, leading to improved outcomes for patients detected at an early stage (3–5). However, some patients still face challenges such as tumor recurrence, metastasis, and mortality (6), which may be related to the characteristics of the solid pulmonary nodules, including ground-glass opacity lesions that are associated with a more favorable prognosis in early-stage NSCLC (7).The significance of tumor-infiltrating immune cells in the treatment and prognosis of NSCLC is progressively gaining recognition (8, 9). Nevertheless, the correlation between phenotypes of TILs and the local prognosis of NSCLC remains inconclusive (8). Consequently, there is a pressing need for a novel and accurate method to predict the immune biological markers of prognosis in NSCLC.

Tumor-infiltrating lymphocytes (TILs) are a heterogeneous group of cells that include T cells, B cells, natural killer (NK) cells, and dendritic cells (10), each with distinct roles in the tumor microenvironment. The presence and phenotype of TILs have been correlated with patient outcomes, suggesting that they could serve as potential biomarkers for prognosis (9, 11–15). Researches have shown that the role of tumor-infiltrating immune cells (TILs) in NSCLC is increasingly recognized for its impact on treatment and prognosis (8, 9). In the context of NSCLC, the density and composition of TILs, including CD3+, CD4+, CD8+, and FOXP3+ lymphocytes, have been studied for their association with patient survival and response to therapy. High levels of CD8+ TILs, for example, have been associated with better survival rates, while FOXP3+ regulatory T cells (Tregs) may have a suppressive effect on immune responses (16, 17).

Besides, it is clear that TILs are not only important in NSCLC but also in other cancer types such as breast cancer (18) and melanoma (19). For instance, in breast cancer, the presence of TILs within tumors has been shown to indicate an immunogenic character, with certain subtypes like triple-negative breast cancer (TNBC) and HER2-positive breast cancer demonstrating a higher number of TILs compared to hormone receptor-positive (HR+) subtypes (18). This suggests that TILs could be a common predictive marker across different cancer types.

The interaction between TILs and the tumor microenvironment is complex and can be influenced by various factors, including the tumor’s ability to evade immune detection and the presence of immunosuppressive cells (20). Understanding these dynamics is crucial for developing effective immunotherapies.

While the correlation between TIL phenotypes and local prognosis in NSCLC remains an area of active research, the body of evidence supporting the prognostic significance of TILs is growing. There is a clear need for further studies to refine the understanding of TIL subtypes and their roles in NSCLC, which will be vital for identifying novel and accurate immune biomarkers for prognostic evaluation and guiding treatment strategies in NSCLC and potentially other cancer types.

We conducted a literature search for articles on the prognostic significance of TILs, CD3+, CD4+, CD8+, and FOXP3+ lymphocytes in NSCLC. Our objective is to furnish precise biomarkers for prognostic evaluation and treatment of NSCLC.

## 2 Materials and methods

The systematic review and meta-analysis conformed to the Preferred Reporting Items for Systematic Reviews and Meta-Analyses (PRISMA) guidelines, as elucidated in **Supplementary Material 1**.

### 2.1 Registration

The research protocol was registered with PROSPERO and assigned the registration number CRD42023468089.

### 2.2 Literature source

All published literature published prior to December 19, 2023 were searched from databases including PubMed, EMBASE, Web of Science, and the Cochrane Library. The search terms applied in PubMed included “Lung Cancer”, “Pulmonary Neoplasms”, “Cancer of the Lung”, “Pulmonary Cancer”, “Tumor Infiltrating Lymphocyte”, “Tumor-Infiltrating Lymphocyte”, “Tumor Derived Activated Cells”. The detailed strategies for comprehensive searches are presented in **Supplementary Material 2**.

### 2.3 Study selection

As inclusion criteria, studies had to report the standard prognostic endpoints of OS and/or disease-free survival (DFS). The target population was all patients with NSCLC who had been tested for levels of TILs or other subtypes including CD3+, CD4+, CD8+, and FOXP3+ lymphocytes. Researches were mandated to be published as original articles and provide ample data necessary for the computation of the hazard ratios (HRs) and 95% CI. To uphold a satisfactory statistical rigor, studies with sample sizes below 30 were excluded. Furthermore, the following reports were excluded: (1) duplicates; (2) case reports; (3) letters, comments, or editorials; (4) reviews and/or meta-analyses; (5) guidelines, notes, or reports; (6) experimental or animal studies; (7) meeting report; (8) clinical trial registration; (9) patients without NSCLC; and (10) survival outcomes such as OS were not reported. The publication year was not restricted.

### 2.4 Data extraction

The following data were extracted from each study: first author’s name, year of publication, country, study design, type of publication, enrollment period, total patient count, patient age, tumor stage, histologic subtype, phenotype of TILs, geographical location, definition of enriched TILs, outcomes observed, intervention measure, and duration of follow-up.

### 2.5 Study quality

The evaluation of literature quality was executed utilizing the Newcastle-Ottawa Quality Assessment Scale (NOS). Two authors, QY and SL, scored each study based on the selection of study population (0 - 4 stars), comparability of study groups (0 - 2 stars), determination of outcomes for cohort studies, and exposure for case-control studies (0 - 3 stars). The total NOS scores ranged from 0 to 9 stars, with 0 indicating the lowest quality and 9 signifying the highest. Within this scale, 0 to 3 stars are indicative of low quality, 4 to 6 stars represent medium quality, and 7 to 9 stars denote high quality. Any discrepancies in the evaluation was addressed through discussion, and if necessary, a senior author (V.S.) was consulted.

### 2.6 Statistical analysis

Statistical analyses were conducted using STATA version 18.0 (MP-Parallel Edition). Given the observed heterogeneity in effect sizes across studies, only the random effects model was utilized for the analysis. The corresponding HR and 95% CI were subsequently summarized. Heterogeneity was assessed employing both the Cochrane Q test and the I² statistic, wherein P < 0.1 or I² > 50% signified noteworthy heterogeneity across the studies. Subgroup analyses and meta-regression was employed to explore the fundamental determinants for the observed heterogeneity. Covariates in this analysis included study quality, tumor location, and population region. Additionally, subgroup analyses were executed utilizing the aforementioned covariates. The Egger test was employed to assess publication bias, with a significance level set at P<0.1.Funnel plot was used to show the associations between HR and standard error for individual studies to assess publication bias. The trim and fill method was proposed to address any detected publication bias.

## 3 Results

### 3.1 Study characteristics

**Figure 1:**
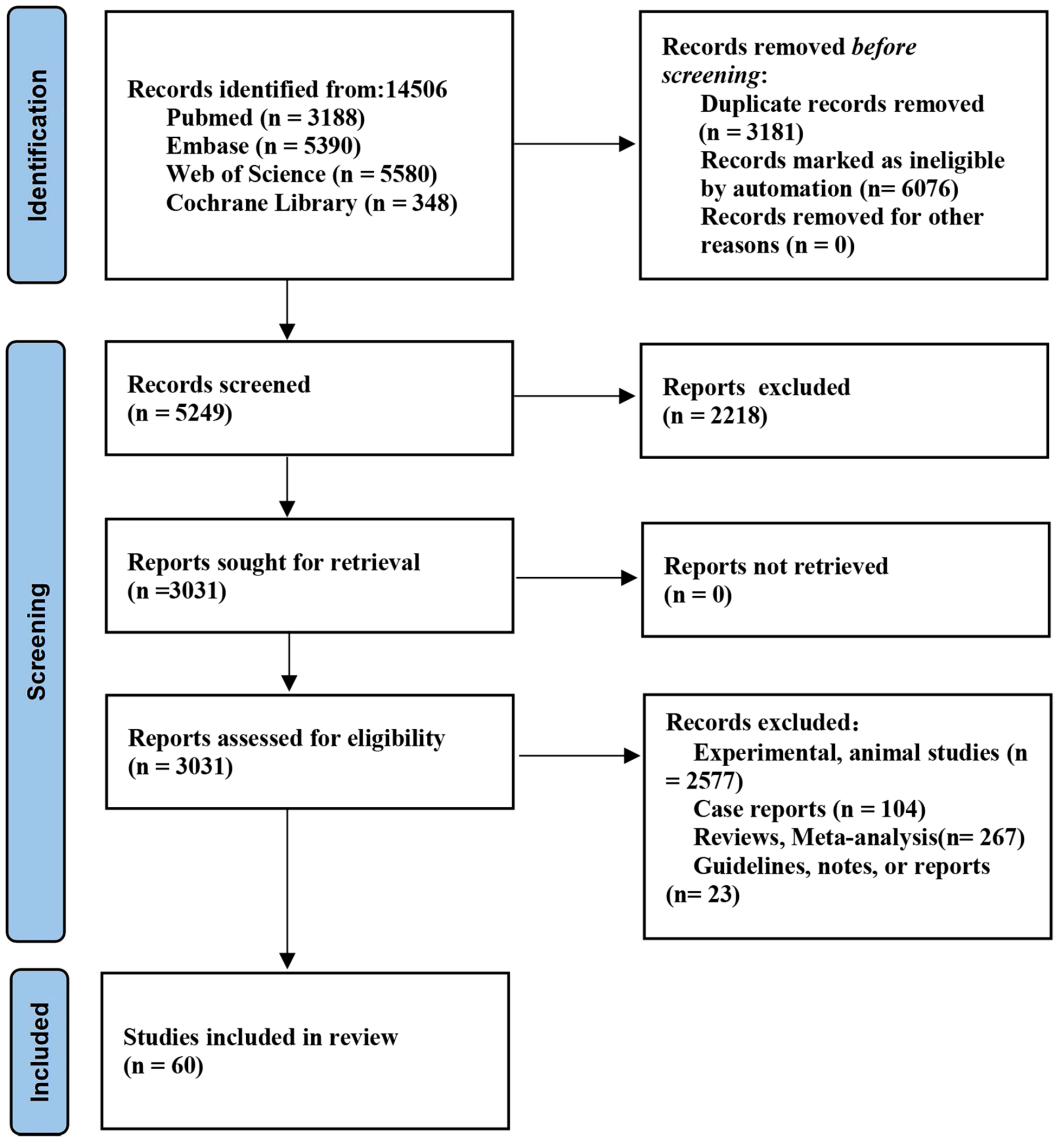
Flowchart of literature screening according to inclusion and exclusion criteria.

Initially, a total of 14,506 papers were identified from databases. Finally, 60 studies (11–13, 21–77) (15829 patients with NSCLC) were eligible (**Figure 1**). The characteristics of the incorporated studies are delineated in **Supplementary Table S1**. The sample size was between 30 and 1546 patients. There were 55 full-text publications and 5 abstracts. The studies were published from 2003 to 2023. 26 studies were conducted in Asia, 20 in Europe, and 8 in North America. 14 studies had moderate quality and 46 high quality (**Supplementary Table S1**).

### 3.2 Patient characteristics

The age of patients ranged from 18 to 96 years. Among the patients, 7040 were diagnosed with adenocarcinoma, 5576 with squamous cell carcinoma, and 3213 presented with an unclear pathological type. Furthermore, there were 9390 patients who were diagnosed at stage I-II, 3751 at stage III-IV, and 2688 whose stage was indeterminate.

#### 3.2.1 CD8+ TILs

##### 3.2.1.1 OS

**Figure 2:**
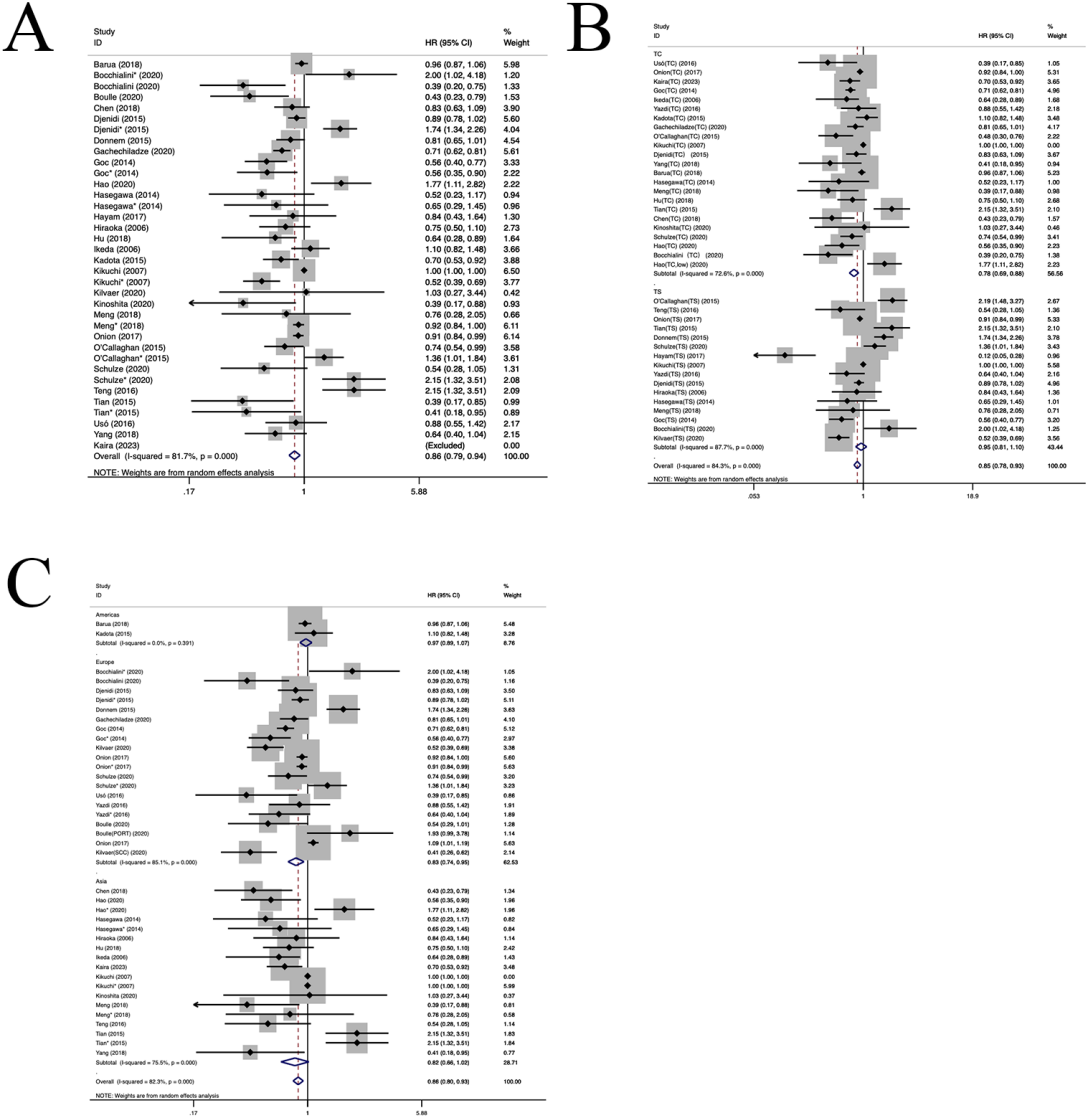
Forest plots of the subgroup analysis of CD8+ TILs on OS in patients with NSCLC. TILs (tumour-infiltrating lymphocytes), OS (overall survival), HRs (hazard ratios), 95% CIs (95% confidence intervals), TC (tumour compartment), TS (tumour stroma), SCC(squamous cell carcinoma), PORT(First had tumour surgical resection followed by treatment with chemotherapy and radiotherapy).* stands for TS. **(A)** Forest plots of the prognostic value of CD8+TILs on OS in patients with NSCLC; **(B)** Forest plots of the prognostic value of different locations of CD8+TILs; **(C)** Forest plots of the prognostic value of different populations of CD8+TILs.

28 publications involving 58 cohorts reported the prognostic significance of CD8+ TIL (11–13, 21–45). As illustrated in **Figure 2**, a positive correlation was observed between a heightened density of CD8+ TILs and enhanced OS in individuals diagnosed with NSCLC (HR=0.86, 95%CI:0.79-0.94, P<0.05, **Figure 2A**). The observed heterogeneity in this relationship was statistically significant (I^2 ^= 81.7%, P<0.001). Subgroup analysis indicated a significant correlation between the presence of CD8+TILs in the TC location and improved OS(HR=0.78, 95%CI:0.69-0.88, P<0.001, **Figure 2B**). In contrast, CD8+TILs in the TS location did not exhibit a significant association with OS(HR=0.95, 95%CI:0.81-1.10, P=0.46, **Figure 2B**). Moreover, subgroup analysis demonstrated a positive correlation between CD8+TILs and enhanced OS in the European population(HR=0.83, 95%CI: 0.74-0.95, P=0.004, **Figure 2C**). However, in the Asian (HR=0.82, 95%CI: 0.66-1.02, P=0.07, **Figure 2C**) and American populations(HR=0.97, 95%CI: 0.89-1.07, P=0.57, **Figure 2C**), CD8+TILs did not yield a significant association with OS. No heterogeneity sources were identified through regression and sensitivity analyses. A notable publication bias was identified within the investigated studies (P=0.011). An iterative method was employed to estimate for the number of missing studies, which was found to be 7 (**Supplementary Figure S1**). Consequently, data from seven virtual studies were incorporated into the meta-analysis. The results did not demonstrate any reversal, thereby confirming the robustness of the pooled outcomes.

##### 3.2.1.2 DFS

**Figure 3:**
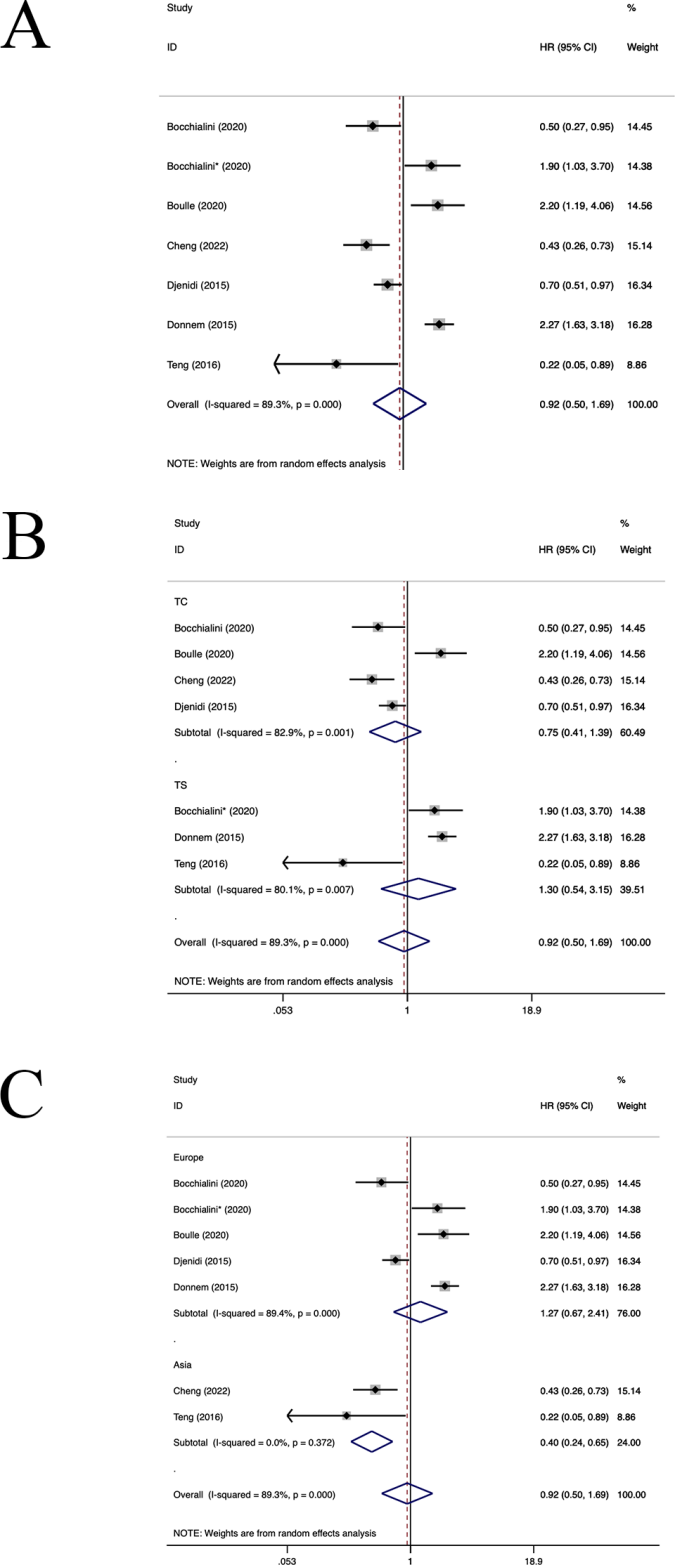
Forest plots of the subgroup analysis of CD8+TILs on DFS in patients with NSCLC. TILs (tumour-infiltrating lymphocytes), DFS (disease-free survival), HRs (hazard ratios), 95% CIs (95% confidence intervals), TC (tumour compartment), TS (tumour stroma).* stands for TS. **(A)** Forest plots of the prognostic value of CD8+TILs on DFS in patients with NSCLC; **(B)** Forest plots of the prognostic value of different locations of CD8+TILs; **(C)** Forest plots of the prognostic value of different populations of CD8+TILs.

Six studies (21, 22, 25, 26, 41, 46) have estimated the prognostic value of CD8+ TILs. As shown in **Figure 3**, a higher density of CD8+ TILs was correlated with an improved DFS in patients with NSCLC (HR=0.92, 95%CI: 0.50-1.69, P>0.05, **Figure 3A**), with significant heterogeneity (I^2 ^= 89.3%, P<0.001). Subgroup analyses revealed no notable difference in DFS between the CD8+TILs in TC location(HR=0.75, 95%CI: 0.41-1.39, P=0.36, **Figure 3B**) and the TS location(HR=1.30, 95%CI: 0.54-3.15, P=0.56, **Figure 3B**). Furthermore, in the Asian population, CD8+TILs were associated with better OS(HR=0.40, 95%CI: 0.24-0.65, P<0.001, **Figure 3C**). However, in the European population (HR=1.27, 95%CI: 0.67-2.41, P=0.46, **Figure 3C**), CD8+TILs did not yield a significant association with DFS. Both regression and sensitivity analyses failed to pinpoint any sources of heterogeneity. The examined studies did not exhibit any significant evidence of publication bias (P=0.556),as well as shown in the funnel plot (**Supplementary Figure S2**).

#### 3.2.2 FOXP3+ TILs

**Figure 4:**
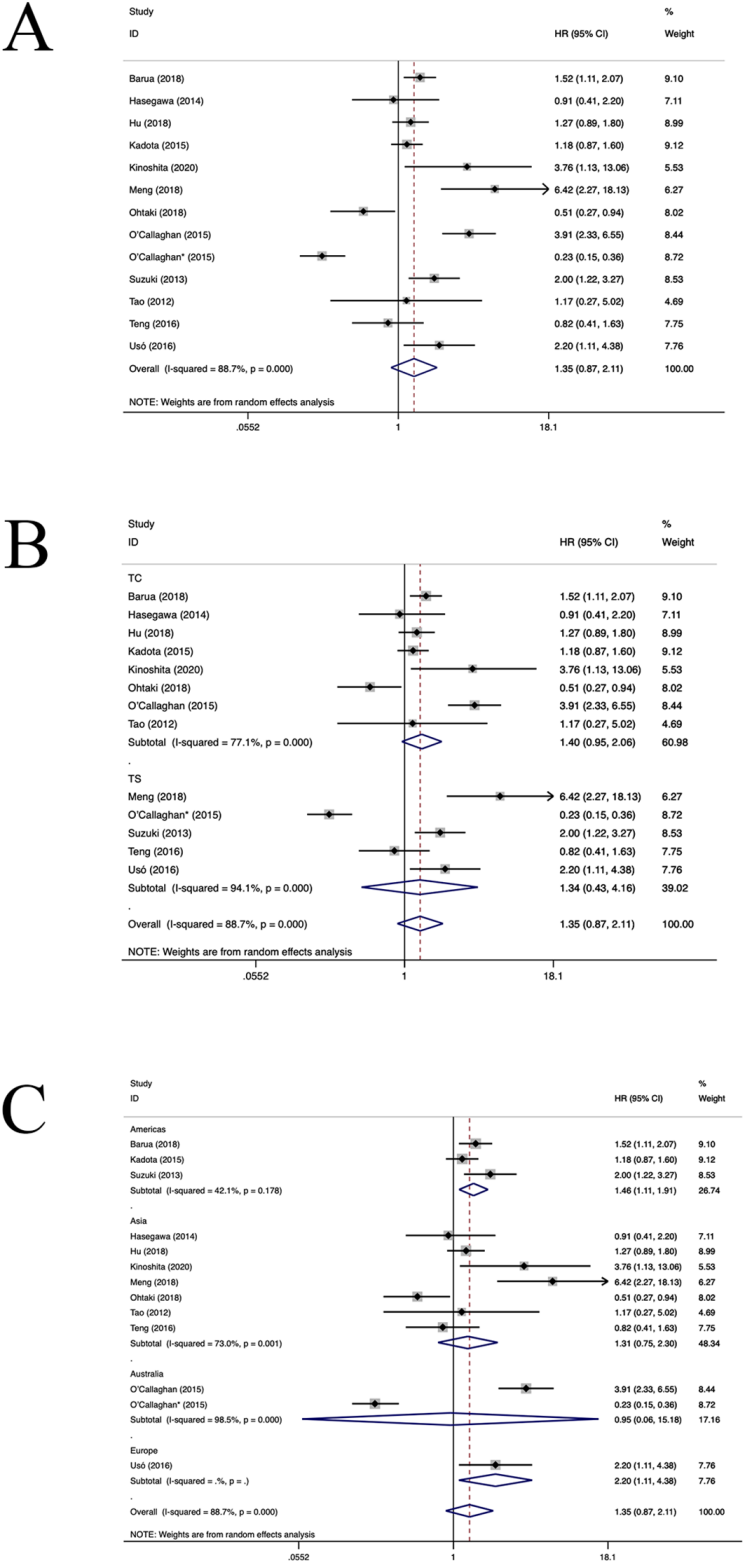
Forest plots of the subgroup analysis of FOXP3+TILs on OS in patients with NSCLC. FOXP3+ (forkhead box P3+),TILs (tumour-infiltrating lymphocytes), OS (overall survival), HRs (hazard ratios), 95% CIs (95% confidence intervals), TC (tumour compartment), TS (tumour stroma). * stands for TS. **(A)** Forest plots of the prognostic value of FOXP3+TILs on OS in patients with NSCLC; **(B)** Forest plots of the prognostic value of different locations of FOXP3+ TILs; **(C)** Forest plots of the prognostic value of different populations of FOXP3+TILs.

Twelve studies (23, 29, 30, 34–37, 40, 41, 43, 47, 48) have estimated the prognostic value of FOXP3+TILs. The aggregated findings illustrated a positive correlation between an elevated density of FOXP3+TILs and shortened OS in patients diagnosed with NSCLC (HR=1.35, 95%CI: 0.87-2.11, P>0.05, **Figure 4A**), with significant aheterogeneity (I^2 ^= 88.7%, P<0.001). Subgroup analyses revealed no notable difference in OS between FOXP3+TILs in TC location (HR=1.40, 95%CI: 0.95-2.06, P=0.09, **Figure 4B**) and the TS location (HR=1.34, 95%CI: 0.43-4.16, P=0.61, **Figure 4B**). In the American and European populations, FOXP3+TILs were associated with poorer OS (American population: HR=1.46, 95%CI: 1.11-1.91, P=0.006, European population: HR=2.20, 95%CI: 1.11-4.38, P=0.02, **Figure 4C**). However, in the Asian and Australian populations (HR=1.27, 95%CI: 0.67-2.41, P=0.46, **Figure 3C**), FOXP3+TILs did not yield a significant association with OS(Asian population: HR=1.31, 95%CI: 0.75-2.30, P=0.34; Australia population: HR=0.95, 95%CI: 0.06-15.81, P=0.97, **Figure 4C**). Regression and sensitivity analyses did not pinpoint any heterogeneity sources. Furthermore, our analysis failed to uncover any significant signs of publication bias across the included studies(P=0.619), which is also reflected in the funnel plot (**Supplementary Figure S3**).

#### 3.2.3 CD3+ TILs

**Figure 5:**
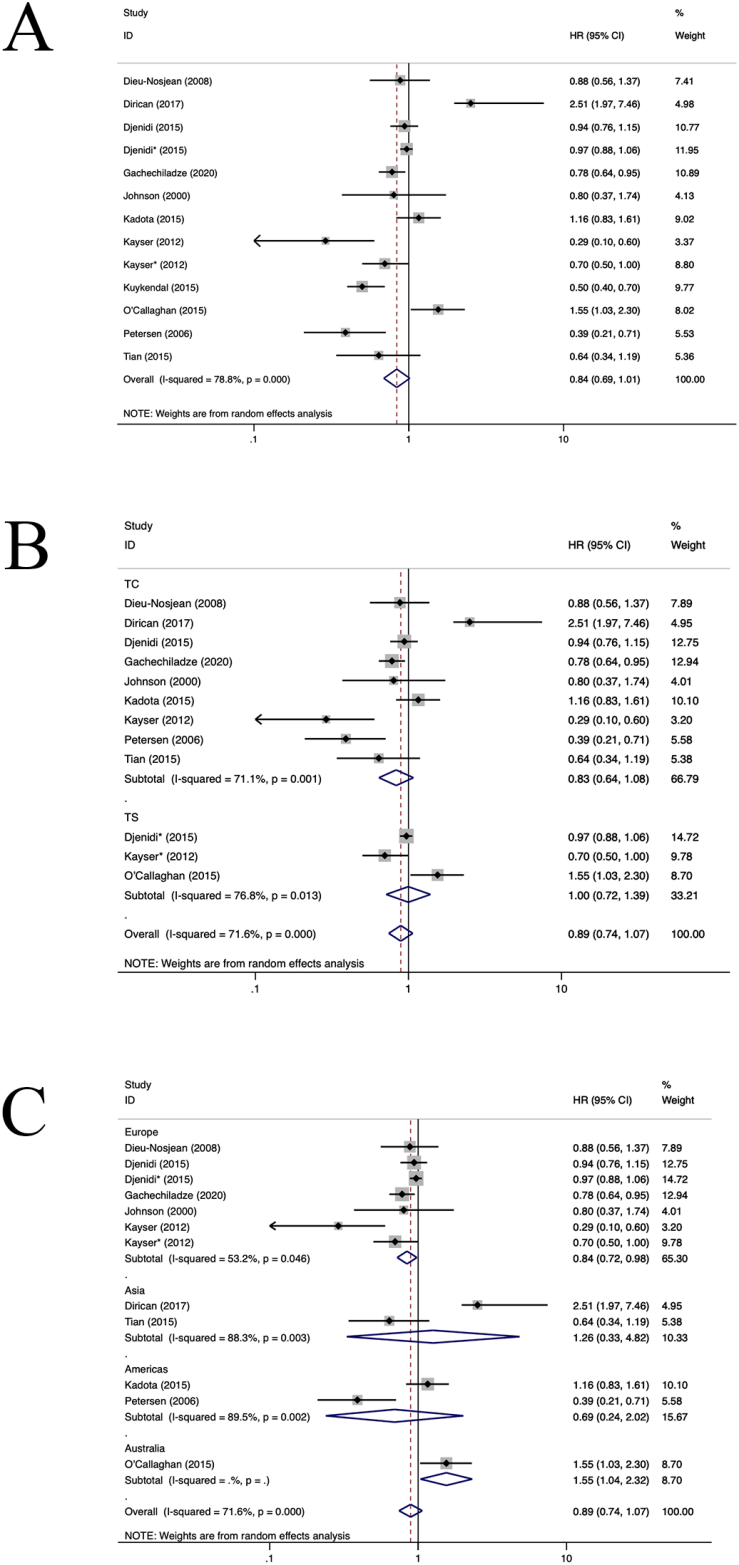
Forest plots of the subgroup analysis of CD3+TILs on OS in patients with NSCLC. TILs (tumour-infiltrating lymphocytes), OS (overall survival), HRs (hazard ratios), 95% CIs (95% confidence intervals), TC (tumour compartment), TS (tumour stroma). * stands for TS. **(A)** Forest plots of the prognostic value of CD3+TILs on OS in patients with NSCLC; **(B)** Forest plots of the prognostic value of different locations of CD3+TILs; **(C)** Forest plots of the prognostic value of different populations of CD3+TILs.

Eleven studies assessed the prognostic significance of CD3+ TILs (26, 27, 35, 37, 42, 49–54). As depicted in **Figure 5**, the pooled analysis revealed that an elevated density of CD3+TILs correlated positively with improved OS for patients diagnosed with NSCLC (HR=0.84, 95%CI: 0.69-1.01, P>0.05, **Figure 5A**), with significant heterogeneity (I^2 ^= x 78.8%, P<0.001). Upon further subgroup analysis, it was observed no notable difference in OS between CD3+TILs in TC location(HR=0.83, 95%CI: 0.64-1.08, P=0.16, **Figure 5B**) and the TS location (HR=1.00, 95%CI: 0.72-1.39, P=0.99, **Figure 5B**). In the European population, CD3+TILs were associated with better OS(HR=0.84, 95%CI: 0.72-0.98, P=0.02, **Figure 5C**). While in the Australian population(HR=1.55, 95%CI:1.04-2.32, P=0.03, **Figure 5C**), CD3+TILs were associated with poorer OS. Moreover, CD3+TILs did not yield a significant association with OS in the American (HR=0.69, 95%CI: 0.24-2.02, P=0.16, **Figure 5C**) and the Asian populations (HR=1.26, 95%CI: 0.33-4.82, P=0.73, **Figure 5C**). Both regression and sensitivity analyses failed to pinpoint any potential sources of heterogeneity. Moreover, our analysis did not reveal substantial indications of publication bias within the selected studies (P=0.397), which is also reflected in the funnel plot (**Supplementary Figure S4**).

#### 3.2.4 TILs

**Figure 6:**
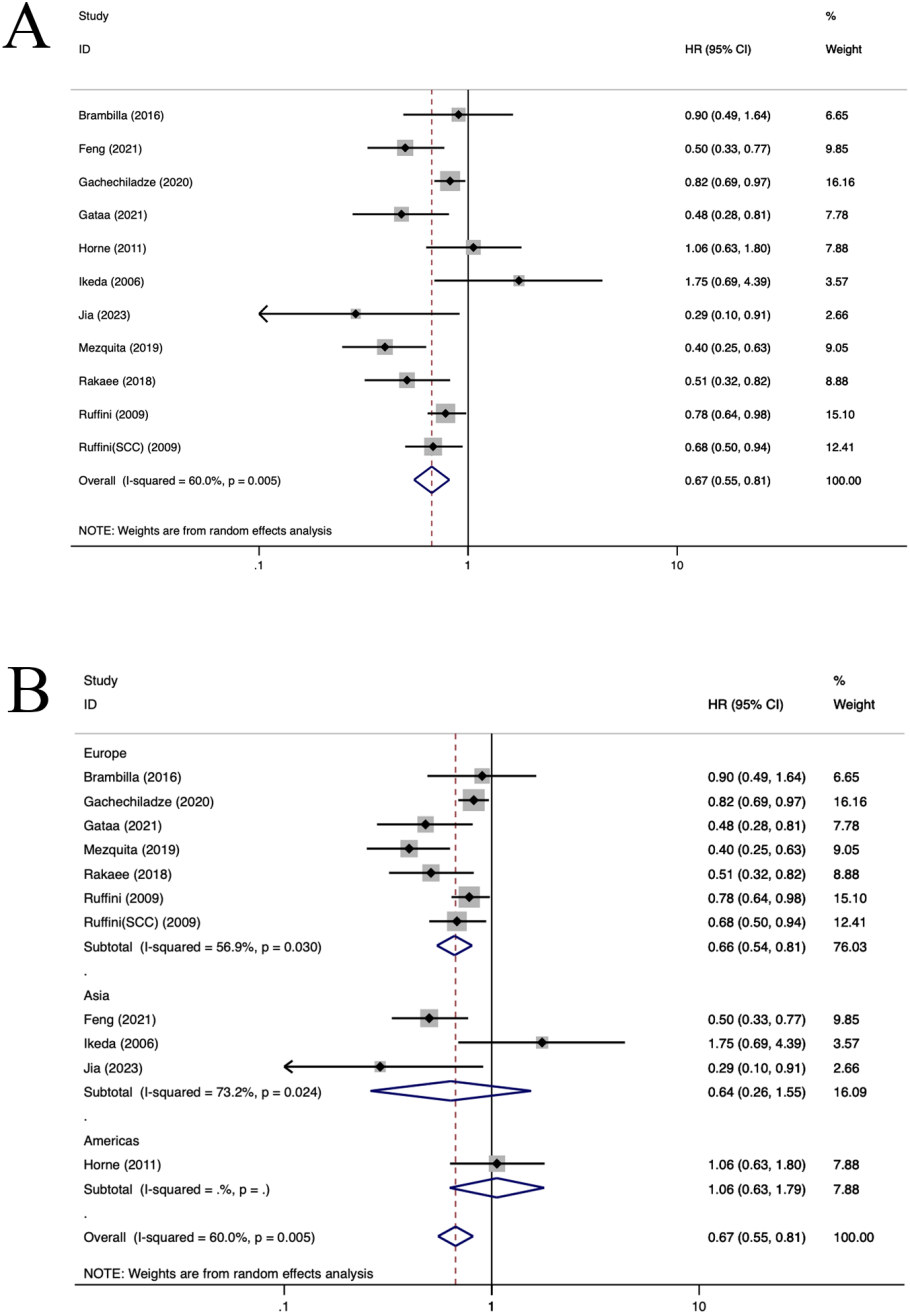
Forest plots of the subgroup analysis of TILs on OS in patients with NSCLC. TILs (tumour-infiltrating lymphocytes), OS (overall survival), HRs (hazard ratios), 95% CIs (95% confidence intervals), SCC(squamous cell carcinoma), TC (tumour compartment), TS (tumour stroma). **(A)** Forest plots of the prognostic value of TILs on OS in patients with NSCLC; **(B)** Forest plots of the prognostic value of different populations of TILs.

Ten selected articles evaluated the prognostic significance of TILs (11, 27, 55–62) (**Figure 6**). The findings demonstrated that an elevated density of TILs correlated with improved OS for patients with NSCLC (HR=0.67, 95%CI: 0.55-0.81, P<0.05, **Figure 6A**). Notably, there was statistically significant heterogeneity observed in the data (I^2 ^= 60.0%, P<0.001). In the European population, TILs+ were correlated with an improved OS (HR=0.66, 95%CI: 0.54-0.81, P<0.001, **Figure 6B**). Both regression and sensitivity analyses failed to pinpoint the source of this observed heterogeneity. Furthermore, no significant publication bias was evident among the included studies (P=0.284), which is also reflected in the funnel plot (**Supplementary Figure S5**).

#### 3.2.5 CD4+ TILs

**Figure 7:**
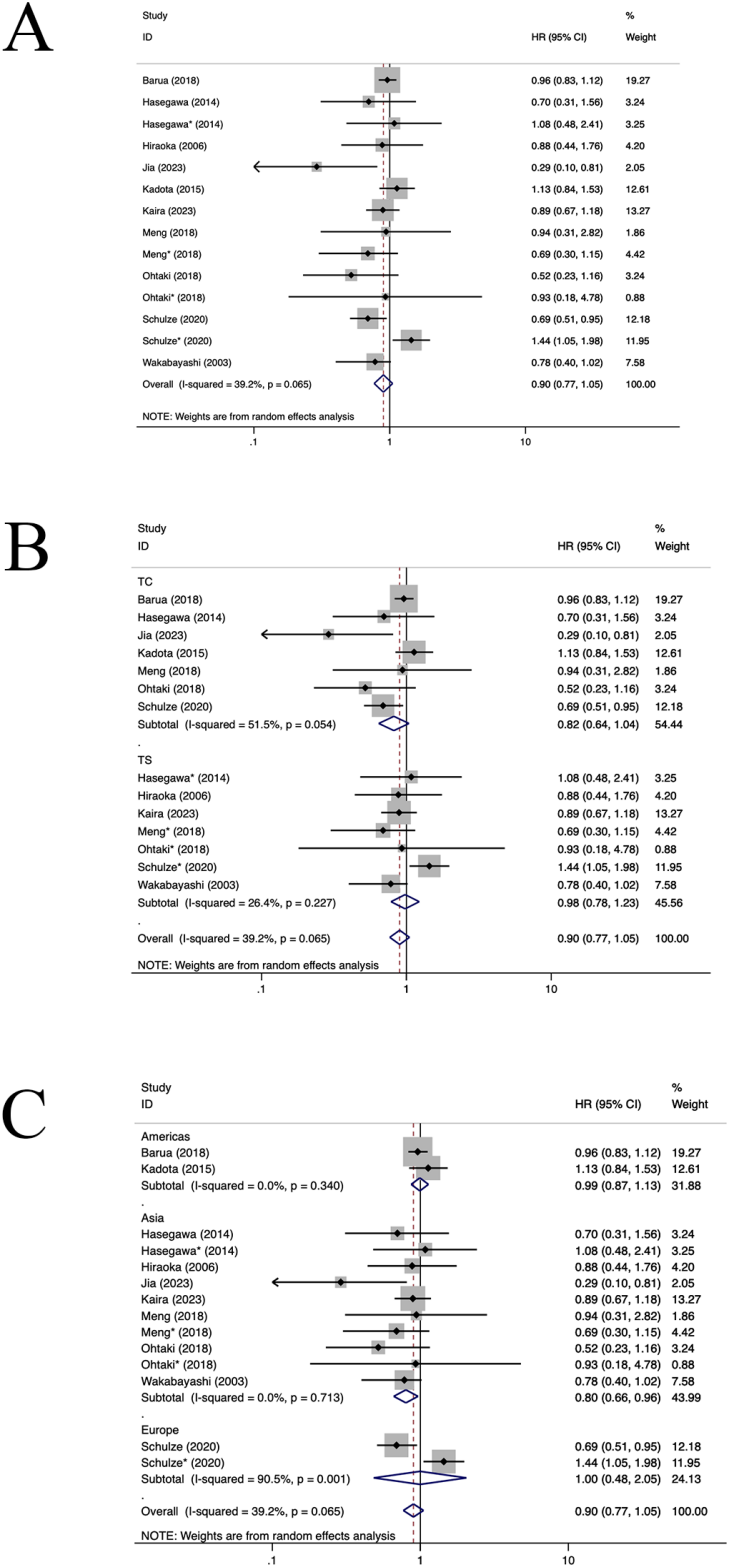
Forest plots of the subgroup analysis of CD4+TILs on OS in patients with NSCLC. TILs (tumour-infiltrating lymphocytes), OS (overall survival), HRs (hazard ratios), 95% CIs (95% confidence intervals), TC (tumour compartment), TS (tumour stroma). * stands for TS. **(A)** Forest plots of the prognostic value of CD4+TILs on OS in patients with NSCLC; **(B)** Forest plots of the prognostic value of different locations of CD4+TILs; **(C)** Forest plots of the prognostic value of different populations of CD4+TILs.

Ten studies (12, 23, 29, 31, 35, 36, 40, 47, 59, 63) assessed the prognostic significance of CD4+ TILs. A positive relationship was observed between a heightened density of CD4+ TILs and enhanced OS in patients with NSCLC (HR=0.90, 95%CI: 0.77-1.05, P>0.05, **Figure 7A**), with significant heterogeneity (I^2 ^= 39.2%, P=0.065). Subgroup analysis indicated that there was no notable difference in OS between CD4+TILs in TC location (HR=0.82, 95%CI: 0.64-1.04, P=0.10, **Figure 7B**) and the TS location(HR=0.98, 95%CI: 0.78-1.23, P=0.86, **Figure 7B**). In Asian, the population of CD4+TILs exhibited a longer OS (HR=0.80, 95%CI: 0.66-0.96, P=0.01, **Figure 7C**). No heterogeneity sources were identified through regression and sensitivity analyses. No statistically significant publication bias was identified within the study populations (P=0.181), which is also reflected in the funnel plot (**Supplementary Figure S6**).

## 4 Discussion

Research has shown that immune cell infiltration, including TILs, plays a significant role in cancer prognosis, with some studies suggesting a link between TILs and patient survival rates in breast cancer (18), melanoma (19) and NSCLC. However, the correlation between TIL subtypes and survival remains a topic of debate (8, 23). Our study, which incorporated a comprehensive analysis of 60 articles and 15,829 NSCLC patients, utilized a random effects model for meta-analysis. Data extracted from 48 articles (11–13, 21–64, 76) were used to evaluate the prognostic role of TILs in NSCLC. Our research indicates that TILs, particularly CD8+, CD3+ and CD4+ TILs, are associated with improved prognosis in NSCLC, supporting the notion that these immune cells contribute to anti-tumor immunity.

Building upon previous meta-analyses that were limited by smaller sample sizes and a lack of analysis on the impact of different regional populations, our study expanded the sample size and conducted a thorough analysis of regional population characteristics and sources of heterogeneity. This approach allowed for a more robust and comprehensive understanding of the relationship between TILs and NSCLC prognosis. Our research demonstrated that both the TILs infiltration location and the region of population were linked with improved prognosis in NSCLC. However, the manifestation of this association varies in intensity across different subtypes of TILs. There is compelling evidence suggesting that TILs are indicative of the adaptive anti-tumor immune response, typically associated with a favorable prognosis (29). This observation aligns with our study’s findings. The subgroup analysis suggested that the spatial distribution of TILs might offer a plausible explanation for the observed heterogeneity. Additionally, we discovered that the prognosis was relatively better among individuals from the European and Asian populations, potentially attributable to racial heterogeneity across different populations. Further investigation is warranted to elucidate this phenomenon.

TIL subtypes predominantly encompass CD3+ TILs, CD4+ TILs, CD8+ TILs, and FOXP3+ TILs. The prognostic significance of CD3+ TILs in NSCLC remains debatable. Some investigations have identified an imbalance in the expression of NKG2D and NKG2A receptors on CD3+ lymphocytes. This imbalance may correlate with poor prognosis, heightened malignancy, and immune evasion in advanced cancer stages, and is implicated in the progression of lung cancer (78). However, existing evidence suggests that the infiltration of augmented CD3+ T cells independently predicts clinical benefit in patients with NSCLC (42, 64). Our study corroborated a superior prognosis associated with CD3+ TILs in NSCLC, despite no statistically significant variance. Consequently, further research is warranted to clarify the function of CD3+ TILs within the immune microenvironment of NSCLC.

Throughout the course of tumor progression, cytotoxic T lymphocytes (CTLs) may exhibit functional impairment or exhaustion, attributable to immune-related tolerance and immunosuppression within the tumor microenvironment. This can lead to adaptive immune resistance (79). The primary anti-tumor immune cells in this environment are CD4+ and CD8+ T cells (80). Prior studies have elucidated that antigen-specific CD4+ T cells contribute to anti-tumor immunity by aiding CD8+T cells (81). We have collated pertinent studies to assess the prognostic significance of CD4+TILs and CD8+TILs in NSCLC. Our findings indicate that CD4+TILs correlate with a favorable prognosis in NSCLC patients with no statistical significance, whereas CD8+TILs are associated with enhanced OS(with statistically significant), aligning with prior research outcomes (82). However, some evidence suggests that tumor-infiltrating CD4+ and CD8+ T cells do not correlate with OS or DFS. In contrast, high stromal infiltration of CD4+ T cells has been associated with improved OS in patients with NSCLC (83, 84). Contradictory conclusions from multiple studies may be attributed to the hierarchical levels of stromal-infiltrating immune cells (85). Further research is required to clarify the prognostic significance of stromal-infiltrating immune cells at different concentrations in NSCLC.

FOXP3, a significant regulatory factor for regulatory T cells, is frequently found to be aberrantly expressed in lung cancer cells (86). Recent studies have increasingly underscored the oncogenic role of FOXP3 in lung cancer. These investigations propose that FOXP3 functions as a co-activator to facilitate the Wnt-β-catenin signaling pathway and induce epithelial-mesenchymal transition, thereby promoting tumor growth and metastasis in NSCLC (16, 17). Additionally, FOXP3 may enhance the invasiveness and migratory capabilities of NSCLC cells by modulating the vascular endothelial growth factor (VEGF), EMT, and Notch1/Hes1 pathways (87). Our research also revealed an association between FOXP3 expression in NSCLC and poor prognosis. This could potentially be attributed to the potential of FOXP3 regulatory T cells to obstruct effective anti-tumor immune responses (88). Grell et al. documented a correlation associating FOXP3 positivity with reduced PFS and OS (89). However, another study suggested that tumor FOXP3 expression held prognostic value and improved survival outcomes in NSCLC (48, 82). The conflicting findings from these studies underscore the necessity for further comprehensive research into the role of FOXP3 in NSCLC.

While our meta-analysis is not the inaugural study to explore the relationship between TILs and NSCLC prognosis, it may yield divergent conclusions from prior research. Our inclusion of 60 articles and 15829 patients substantially expands upon sample sizes in previous studies. Notably, neither subgroup regression analysis nor sensitivity analysis were conducted in earlier investigations. Our findings underscore the prognostic significance of TILs in NSCLC, with a more pronounced effect observed among the European and Asian populations. Specifically, CD4+TILs in the Asian population, CD3+TILs in the European population, CD8+TILs in the Asian and European populations emerged as favorable prognostic biomarkers for NSCLC. Moreover, CD8+TILs demonstrated a marked survival advantage specifically when they infiltrated the tumor. This was evidenced by a survival benefit in the European population, as well as a significant enhancement in DFS for NSCLC in the Asian population. In contrast, the prognosis of FOXP3+TILs was not significantly improved in NSCLC across all ethnicities, and even worse in the American and European populations. These findings suggest that distinct TIL subtypes exert varied prognostic roles in NSCLC patients, rendering them viable biological markers for treatment strategies, prognostic evaluations, and recurrence or metastasis monitoring.

Despite the comprehensiveness of this study, certain limitations must be acknowledged. Primarily, significant heterogeneity was observed in most of the pooled results, with no substantial differences identified through subgroup analysis or meta-regression. This heterogeneity could potentially be attributed to individual patient variations within the clinical studies, including the varying quality levels of these studies, and the differing assessment criteria for TIL density. Additionally, selective reporting of positive data in some studies could compromise the authenticity of the findings. Addressing these limitations in future research will be crucial for refining our understanding of the complex interplay between TILs and NSCLC prognosis.

## Conclusions

Our research indicates that TILs, particularly CD8+, CD3+ and CD4+TILs, are associated with improved prognosis in NSCLC, supporting the notion that these immune cells contribute to anti-tumor immunity. The conflicting conclusions from various studies highlight the need for further research to elucidate the prognostic significance of TIL subtypes in NSCLC and their potential as biological markers for treatment strategies and prognostic evaluations.

## Data Availability

The original contributions presented in the study are included in the article/**Supplementary Material**. Further inquiries can be directed to the corresponding authors.
