# Supplementary material for: Prognostic implications of tumor-infiltrating lymphocytes in non-small cell lung cancer: a systematic review and meta-analysis
Source: Front Immunol. 2024 Sep 20;15:1476365. doi: 10.3389/fimmu.2024.1476365 (PMC11449740; doi:10.3389/fimmu.2024.1476365)
Supplement: Supplementary file 2 [file DataSheet2.docx]

**Supplementary Material 2**

**Search strategies for** **Tumor-infiltrating Lymphocytes in** **Non-small Cell Lung Cancer****Preliminary searches strategies prepared 19 December 2023**

**Pubmed**

((((((((Tumor Infiltrating Lymphocyte) OR (Tumor Infiltrating Lymphocytes)) OR (Tumor-Infiltrating Lymphocytes)) OR (Tumor-Infiltrating Lymphocyte)) OR (Tumor-Derived Activated Cells)) OR (Tumor Derived Activated Cells)) OR (Tumor-Derived Activated Cell)) OR (Tumor Derived Activated Cell)) AND ((“Lung Neoplasms”[Mesh]OR“Carcinoma, Non-Small-Cell Lung”[Mesh]) OR (((((((((((Pulmonary Neoplasms) OR (Lung Neoplasm)) OR (Lung Cancer)) OR (Pulmonary Cancer)) OR (Cancer of the Lung)) OR (Non-Small-Cell Lung)) OR (Pulmonary Neoplasm)) OR (Cancer of Lung)) OR (Lung Cancers)) OR (Pulmonary Cancer)) OR (Pulmonary Cancers)))

**Cochrane Library**

#1 MeSH descriptor: [Lung Neoplasms] explode all trees

#2 MeSH descriptor: [Lymphocytes, Tumor-Infiltrating] explode all trees

#3 #1 AND #2

#4 (Lung Cancer):ti,ab,kw OR (Pulmonary Cancer):ti,ab,kw OR(Cancer, Lung):ti,ab,kw OR (Cancer of the Lung):ti,ab,kw

#5 (Cancer of the Lung):ti,ab,kw OR(Pulmonary Neoplasms):ti,ab,kw OR (Lung Neoplasm):ti,ab,kw

#6 #4 or #5

#7 (Tumor-Derived Activated Cell):ti,ab,kw OR(Tumor Derived Activated Cell):ti,ab,kw OR(Tumor Infiltrating Lymphocytes):ti,ab,kw OR (Tumor-Infiltrating Lymphocytes):ti,ab,kw OR(Tumor Infiltrating Lymphocyte):ti,ab,kw

#8 (“tumor Infiltrating Lymphocyte”):ti,ab,kw

#9 #7 or #8

#10 #6 and #9

**Embase**

#1. lung cancer/exp

#2. tumor associated leukocyte/exp

#3. broncho-pulmonary cancer/exp OR bronchopulmonary cancer/exp OR cancer of the lung/exp OR cancer, lung /exp OR carcinogenesis of the lung /exp OR lung malignancies/exp OR lung malignancy/exp OR malignancies of the lung/exp OR malignancy of the lung/exp OR malignant lung neoplasm/exp OR malignant lung tumor/exp OR malignant neoplasm of the lung/exp OR malignant tumor of the lung/exp OR pulmonary cancer/exp OR pulmonary malignancies/exp OR pulmonary malignancy/exp

#4. cancer associated leucocyte/exp OR cancer associated leucocyte/exp OR cancer associated leukocyte/exp OR cancer associated lymphocyte/exp OR intratumoral leukocyte/exp OR intratumoral lymphocyte/exp OR intratumoural lymphocyte/exp OR lymphocyte,tumor-infiltrating/exp OR lymphocytes,tumor-infiltrating/exp OR tumor associated lymphocyte/exp OR tumor infiltrating leukocyte/exp OR tumor infiltrating lymphocyte/exp OR tumor infiltrating lymphocytes/exp OR tumour associated leucocyte/exp OR tumour associated lymphocyte/exp OR tumour infiltrating leukocyte/exp OR tumour infiltrating leukocytes/exp OR tumour infiltrating lymphocyte/exp OR tumour infiltrating lymphocytes

#5. #1 OR # 3

#6. #2 OR #4

#7. #5 AND #6

**Web of Science**

#5 #3 AND #4

#4 TI=(random* or blind* or placebo* or meta-analys* or trial*) OR TS=(random* or blind* or placebo* or meta-analys*)

#3 #2 AND #1

#2 TS=(Tumor Infiltrating Lymphocyte or Tumor Infiltrating Lymphocytes or Tumor-Infiltrating Lymphocytes or Tumor-Infiltrating Lymphocyte or Tumor-Derived Activated Cells)

#1 TS=(Lung cancer OR Pulmonary Neoplasms OR Lung Neoplasm OR Pulmonary Cancer OR Cancer of the Lung OR Non-small Cell Lung)
