# Supplementary figures and images for: Prognostic implications of tumor-infiltrating lymphocytes in non-small cell lung cancer: a systematic review and meta-analysis
Source: Front Immunol. 2024 Sep 20;15:1476365. doi: 10.3389/fimmu.2024.1476365 (PMC11449740; doi:10.3389/fimmu.2024.1476365)

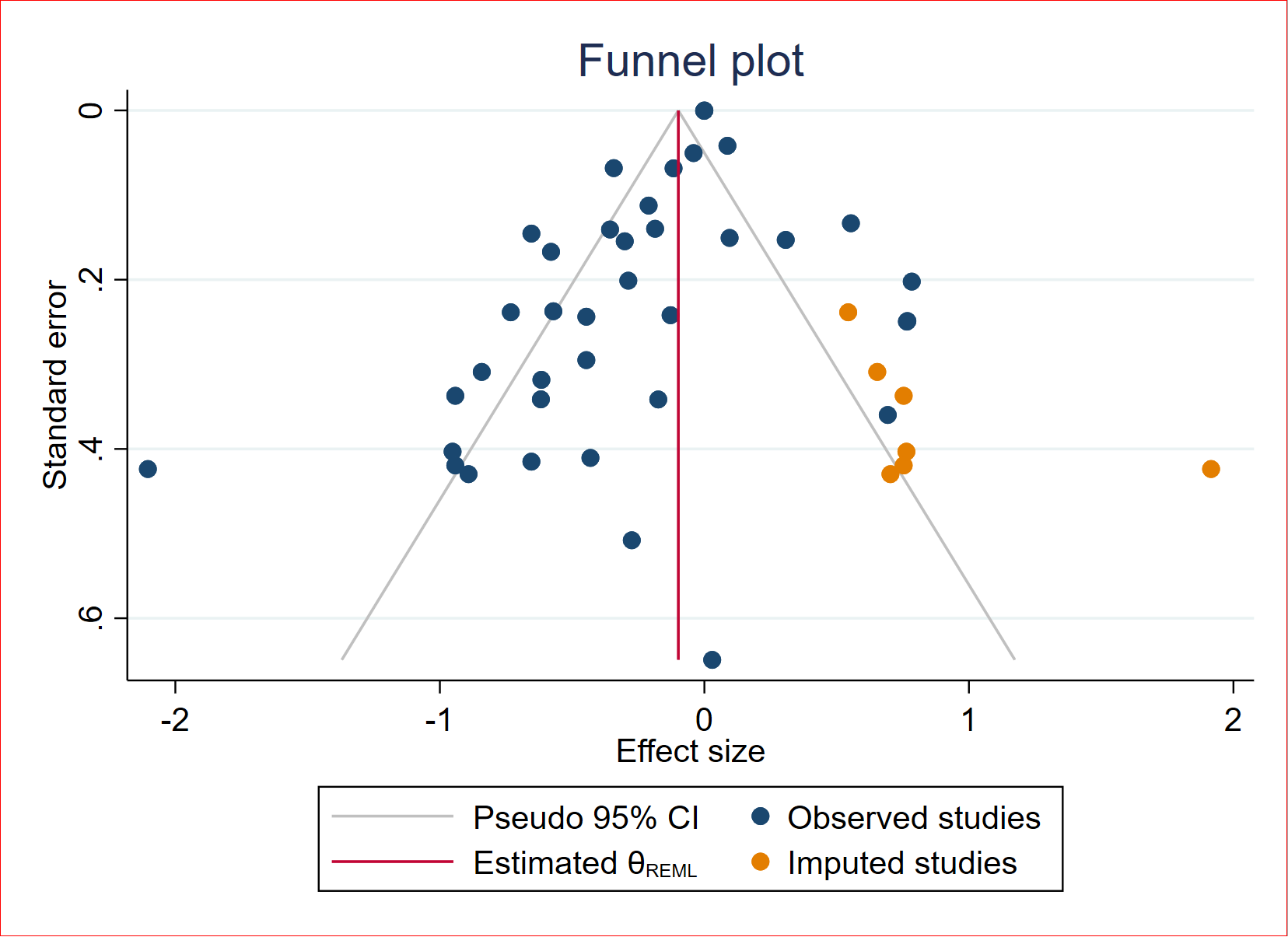

Supplement: Supplementary file 3 [file Image1.jpg]

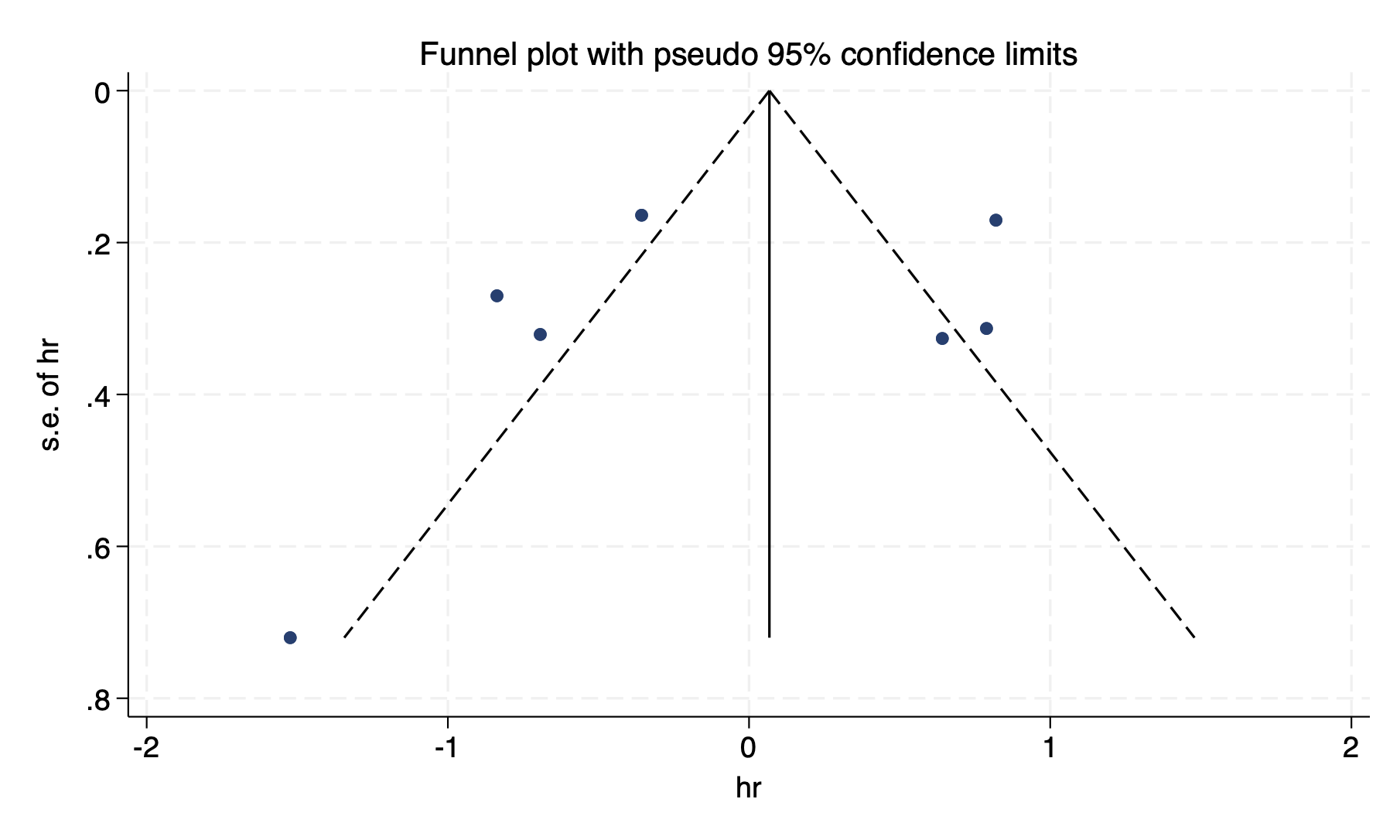

Supplement: Supplementary file 4 [file Image2.tif]

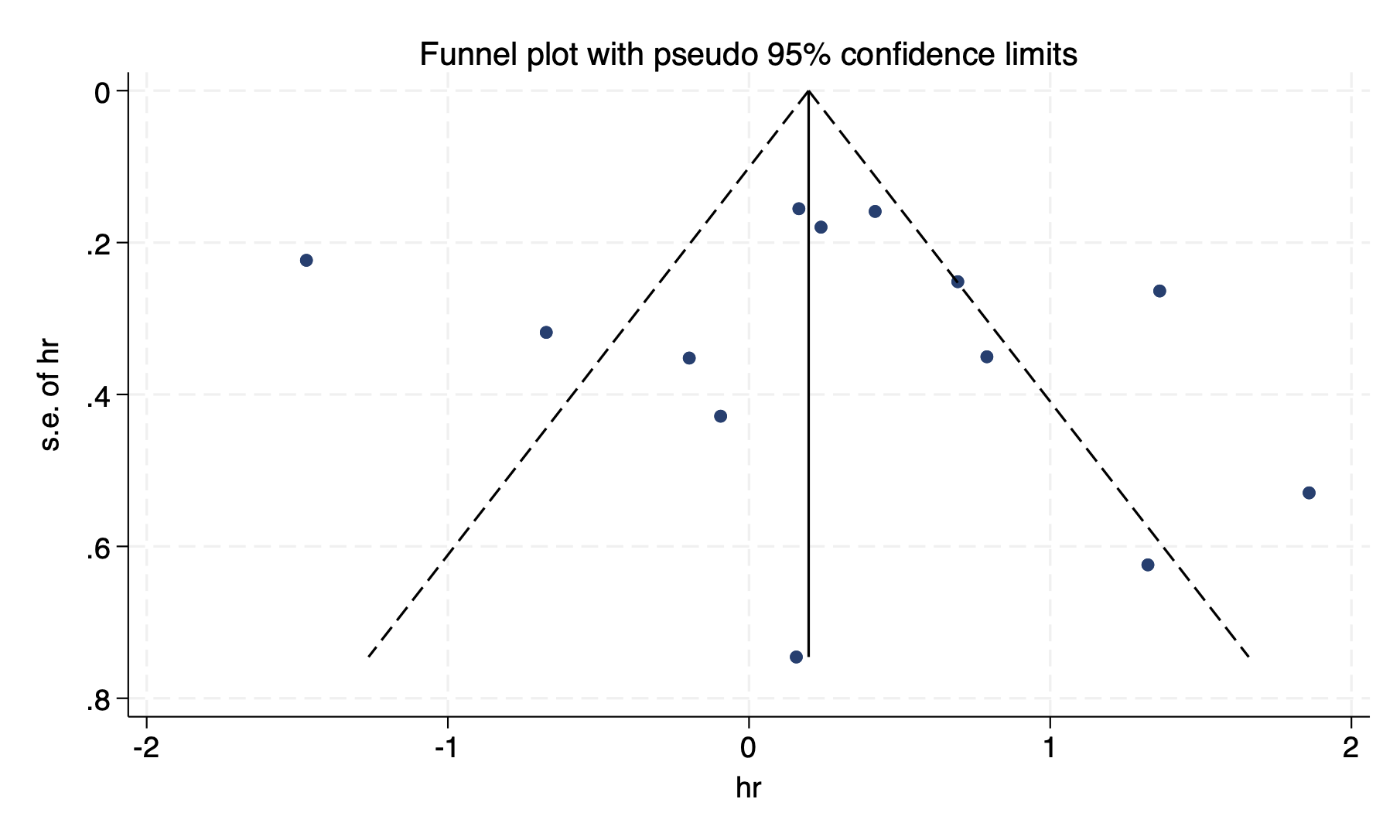

Supplement: Supplementary file 5 [file Image3.tif]

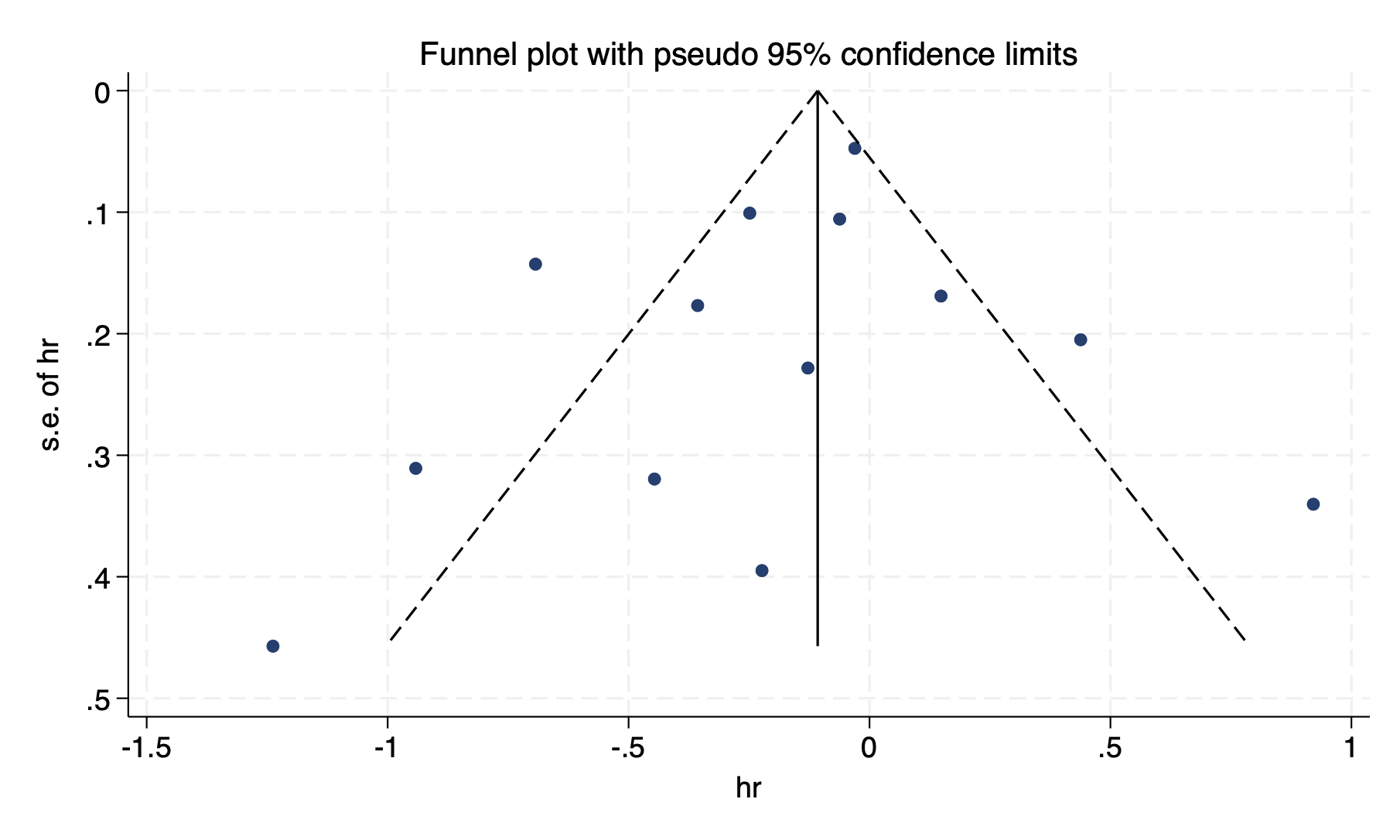

Supplement: Supplementary file 6 [file Image4.tif]

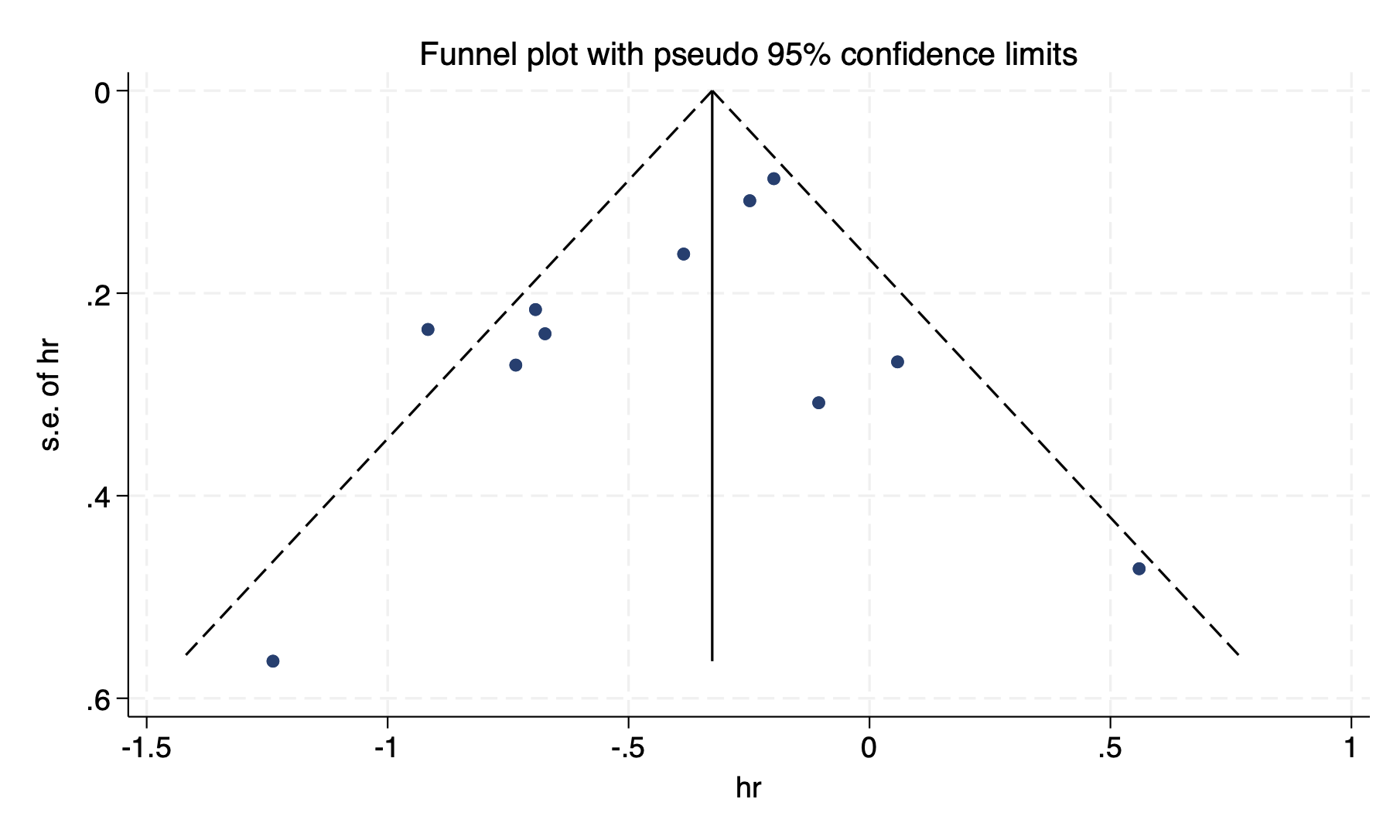

Supplement: Supplementary file 7 [file Image5.tif]

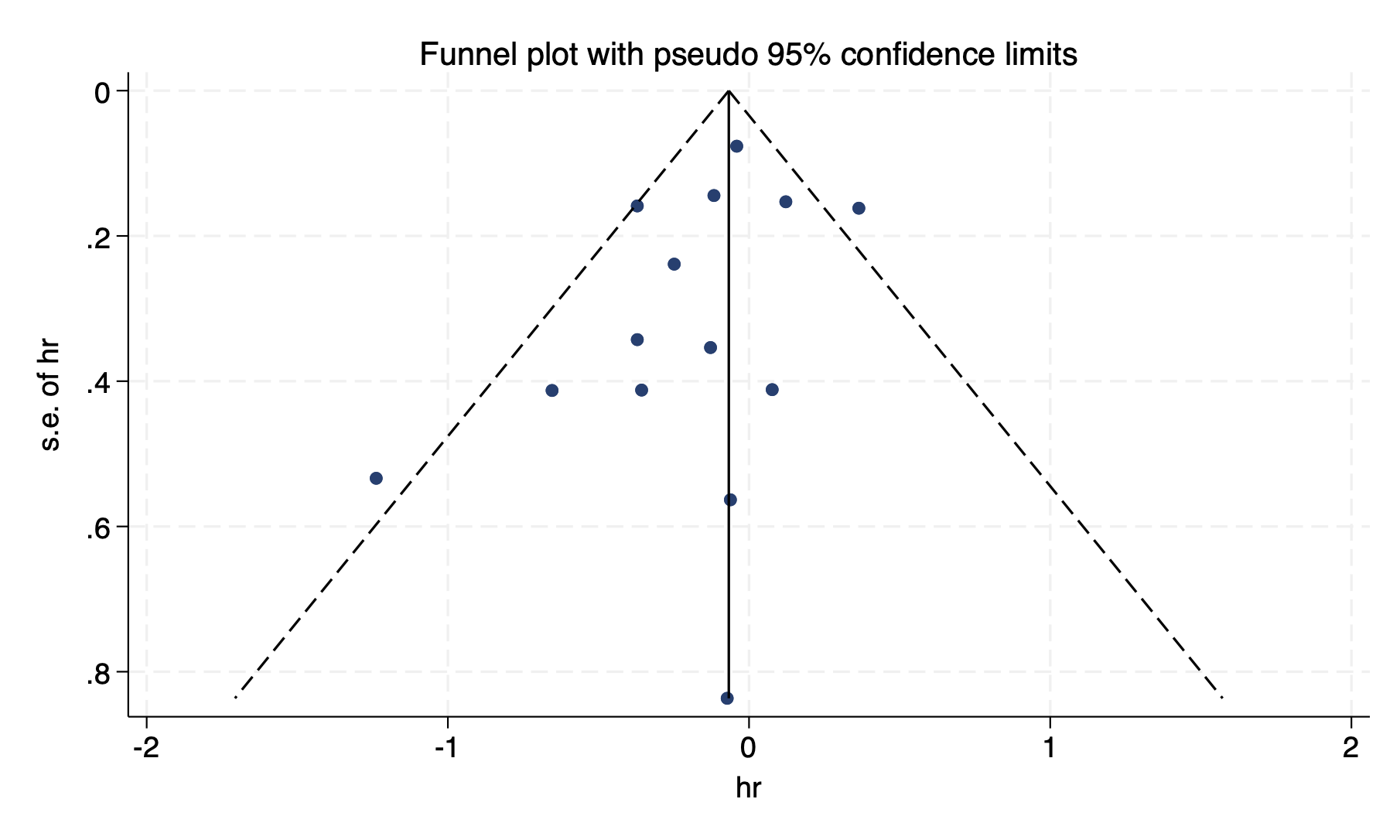

Supplement: Supplementary file 8 [file Image6.tif]
