# Supplementary material for: Prognostic implications of tumor-infiltrating lymphocytes in non-small cell lung cancer: a systematic review and meta-analysis
Source: Front Immunol. 2024 Sep 20;15:1476365. doi: 10.3389/fimmu.2024.1476365 (PMC11449740; doi:10.3389/fimmu.2024.1476365)
Supplement: Supplementary file 9 [file Table1.docx]

**Supplementary Table S1. Study characteristics of the included articles.**

| **Study** | **Country** | **Study design** | **Type of publication** | **Enrollment period** | **No. total Pts. (male,%)** | **Age (years)** | **Tumor stage** | **Histologic subtype** | **TILs phenotype** | **Location** | **Definition of rich TILs** | **Outcomes** | **Intervening measure** | **Follow-up duration (months)** | **NOS score** |
| --- | --- | --- | --- | --- | --- | --- | --- | --- | --- | --- | --- | --- | --- | --- | --- |
| Al-Shibli  (2008) | Norway | Cohort Study | Full-text | 1990 to 2004 | 335(75.5%) | median: 67 (range: 28-85) | I: 212(63.2%) II: 91 (27.2%) III: 32 (9.6%) | SCC: 191 (57.0%) ADC: 95 (28.4%) Others: 49(14.6) | CD4 CD8 CD20 | TC  TS | CD4≥5% in TC,  CD4≥25% in TS CD8>5% in TC,  CD8>50% in TS CD20≥1% in TC or TS | DSS | Only Surgery(82%) Surgery+RT(18%) | median: 96 (range: 10–179) | 8 |
| Bocchialini  (2020) | Italy | Cohort Study | Full-text | 2011 to 2015 | 97(75.3%) | mean: 68.7 (range: 54-84) | I: 37(38.1%) II: 31 (32%) III: 29 (29.9%) | SCC: 58 (59.8%) ADC: 39(40.2%) | CD3 CD8 | TC  TS | NA | DFS OS | Surgery(100%) | median: 48 | 8 |
| Boulle  (2020) | France | Cohort Study | Full-text | 2002 to 2015 | 113(61.9%) | median: 61.5 (range: 39-82) | IIIA: 69(61.1%) IIIB: 44(38.9%) | SCC: 28(24.8%) ADC: 70(61.9%) Others: 15(13.3%) | CD8 | TC  TS | CD8+ | OS | Surgery+PORT(78.8%) DRC(21.2%) | median: 29.8 (range: 0.8–149.6) | 8 |
| Barua  (2018) | United States | Cohort Study | Full-text | 1998 to 2009 | 120(43.3%) | median: 63 (range: 34-89) | I: 5 (4.2%) II: 13 (10.8%) III: 102 (85.0%) | SCC: 35(29.2%) ADC: 72(60.0%) Others: 13(10.8%) | CD4 CD8 FOXP3 | TC | NA | OS | Only Surgery(73.3%)Surgery+ACT(26.7%) | NA | 7 |
| Brambilla  (2016) | France | Cohort Study | Full-text | 1994 to 2003 | 1546(76%) | mean: 59.3 | I: 722 (46.7%) II: 537 (34.7%) III: 283 (18.3%) NA: 4(3%) | SCC: 707 (46%) ADC: 634 (41%) Others: 205(13%) | NA | TS | TIL≥50% | OS | No CT: 767 (50%) CT: 779 (50%) | median: 5.4  (range: 0.1-11.3) | 7 |
| Chen  (2018) | China | Cohort Study | Full-text | 2000 to 2016 | 100(86%) | mean: 57 | I/II: 44(44.0%) III/IV: 56(56.0%) | LSC: 100(100%) | CD8 | TC | CD8+ | OS | Surgery(100%) | median: 27.4 (range: 1.0–129.0) | 7 |
| Cheng  (2022) | China | Cohort Study | Full-text | 2013 to 2016 | 180(94.4%) | median: 63  (range: 57-68) | I/II: 135(75.0%) III: 45(25.0%) | SCC: 180(100%) | CD8 | TC | CD8+ | DFS OS | Surgery(100%) | median: 105 | 8 |
| Dieu-Nosjean(2008) | France | Cohort Study | Full-text | 1998 to 2002 | 74(81.1%) | mean: 64  (range: 41-79) | I: 62(83.8%) II: 12 (16.2%) | SCC: 28 (37.8%) ADC: 46 (62.2%) | CD3 CD20 CD45 | TC | CD3+ CD20+ CD45+ | OS/DSS/DFS | Surgery(100%) | ≥48 | 8 |
| Donnem  (2015) | Norway | Cohort Study | Abstract | 1990 to 2004 | 797(NA) | median: 65.2  (range: 27.5-84.7) | NA | NA | CD8 | TS | CD8+＞50% | DFS/OS/DSS | Surgery(100%) | median: 65.5  (range 34.9-34.0) | 7 |
| Dirican  (2017) | Turkey | Cohort Study | Full-text | 2013 to 2014 | 30(83.3%) | range: 46–82 | IIIB: 11(36.7%) IV: 19(63.3%) | SCC: 17(56.7%) ADC: 13(43.3%) | CD3 CD5 | TC  TS | ≥75% | OS | CT(70%) | NA | 7 |
| Djenidi  (2015) | France | Cohort Study | Full-text | 1995 to 2002 | 101(68.3%) | median: 66.1 (range: 40.5-83.6) | I: 101(100%) | SCC: 43(42.6%) ADC: 45(44.5%) Others: 13(12.9%) | CD3 CD8 CD103 | TC  TS | CD3+ CD8+ CD103+ | OS/DFS | Surgery(100%) | median: 75.6 | 8 |
| Feng  (2021) | China | Cohort Study | Full-text | 2005 to 2012 | 288(65%) | median: 59 (range: 22-86) | IIIA: 288(100%) | SCC: 87(30.2%) ADC: 171(59.4%) Others: 30(10.4%) | CD3 CD8 CD45 | TC | >25% | OS  DMFS | Surgery(100%) Surgery+ACT: 247(85.8%) | median: 36.9 (range: 4.4–132) | 8 |
| Gachechiladze(2020) | Czech | Cohort Study | Full-text | 1993 to 2011 | 1115(66.9%) | median: 64 (range: 18-87) | I: 430(38.6%) II: 333 (29.9%) III: 352 (31.5%) | SCC: 474(42.5%) ADC: 559(50.1%) Others: 82(7.4%） | CD3 CD8 TILs | TC | NA | OS/PFS | Surgery(100%) | NA | 6 |
| Goc(2014) | France | Cohort Study | Full-text | 2001 to 2004 | 376(80%) | mean: 62 (range: 19-83) | I: 167(44.4%) II: 101 (26.9%) III: 104 (27.7%) IV: 2(0.5%) NA: 2(0.5%) | SCC: 111(29.5%) ADC: 241(64.1%) Others: 24(6.4%) | CD8 | TC  TS | CD8+ | OS | Surgery(100%) | ≥90 | 8 |
| Gataa  (2021) | France | Cohort Study | Full-text | 2009 to 2017 | 410(66.6%) | NA | NA | SCC: 78(19%) Non-SCC: 332(81%) | NA | TS | TIL≥10% | OS  PFS  ORR  DCR | ICI: 221(53.9%) CT: 189(46.1%) | median: 11 | 7 |
| Hayam  (2017) | Egypt | Cohort Study | Full-text | 2014 to 2016 | 50(82%) | median: 55  (range: 25-77) | III: 29(58.0%) IV: 21(42.0%) | SCC: 20 (40%) ADC: 30 (60%) | CD8 | TS | >50% | PFS  OS | CT(100%) | median: 10  (range: 5-19) | 7 |
| Hao(2020) | China | Cohort Study | Full-text | 2013 to 2016 | 192(70.3%) | median: 62 (range: 35-84) | I: 54(28.0%) II: 16 (8.5%) III: 102 (53.0%) IV: 20(10.4%) | SCC: 83(43.2%) ADC: 109(56.8%) | CD8 FOXP3 | TC | CD8+ FOXP3+ | OS | Surgery: 81(42.2%) Surgery+ACT: 111 (57.8%) | NA | 6 |
| Hasegawa  (2014) | Japan | Cohort Study | Full-text | 2007 to 2008 | 67(65.7%) | mean: 68.4 (range: 45-83) | I: 47(70.2%) II: 7 (10.4%) III: 13 (19.4%) | SCC: 16(23.8%) ADC: 45(67.2%) Others: 6(9.0%) | CD4 CD8 FOXP3 | TC  TS | CD4+ CD8+ FOXP3+ | OS | Surgery(100%) | median: 21.3 | 7 |
| Hiraoka  (2006) | Japan | Cohort Study | Full-text | 1994 to 1996 | 109(65%) | mean: 63 (range: 39-80) | I: 66(60.5%) II-III: 43(39.5%) | SCC: 40(36.7%) ADC: 58(53.2%) Others: 11(10.1%) | CD4 CD8 | TC  TS | ≥mean | OS | Surgery(100%) | ≥60 | 8 |
| Horne  (2011) | United States | Cohort Study | Full-text | 2000 to 2009 | 273(47.6%) | mean: 68 (range: 40-86) | I: 273(100%) | SCC: 94(34.4%) ADC: 147(53.8%) Others: 32(11.8%) | NA | TC  TS | TILs+ | RFS | Surgery(100%) | NA | 7 |
| Hu(2018) | China | Cohort Study | Full-text | 2007 to 2011 | 90(54%) | median: 61.5  (range: 55-71) | I: 29(32.2%) II: 32 (35.6%) III: 28 (31.1%) IV: 1(1.1%) | ADC: 90(100%) | CD8 FOXP3 CD45 | TC  LS | Final score>6 | OS/DFS | Surgery(100%) ACT: 73(81%) | median: 46  (range: 1-121) | 8 |
| Ikeda  (2006) | Japan | Cohort Study | Full-text | 1996 to 1998 | 83(78.3%) | NA | I: 40(48.2%) II: 43(51.8%) | SCC: 23(27.7%) ADC: 49(59.0%) Others: 11(13.3%) | TIL CD8 | TC | TIL≥50 CD8≥5 | OS | Surgery(100%) | NA | 6 |
| Jia(2023) | China | Cohort Study | Full-text | 2013 to 2019 | 89(70.8%) | median: 64 | I: 12(13.5%) II: 10(11.2%) III: 67(75.3%) | SCC: 37(41.6%) ADC: 50(56.2%) Others: 2(2.2%) | CD4 CD8 | TC | NA | OS | NCT+Surgery(100%) | median: 39.8 | 8 |
| Johnson  (2000) | UK | Cohort Study | Full-text | 1980 to 1992 | 95(62.1%) | NA | I: 54(56.8%) II: 17(17.9%) III: 20(21.1%) NA: 4(4.2%) | NA | CD3 CD8 CD57 CD68 | TC | CD3≥10/HPF CD8≥10/HPF CD57+ CD68+ | OS | Surgery(100%) | NA | 7 |
| Kaira  (2023) | Japan | Cohort Study | Full-text | 2017 to 2021 | 107(86.9%) | median: 71 | III: 11 (10.2%) IV: 77(72.0%) NA: 19(17.8%) | SCC: 45 (42.1%) ADC: 52(45.6%) Others: 10(9.3%) | CD4 CD8 FOXP3 | TC  TS | ≥median | PFS  OS | ICI(100%) | median: 12 | 8 |
| Kilic  (2009) | United States | Cohort Study | Full-text | 2002 to 2005 | 219(45.7%) | NA | IA: 131(59.8%) IB: 88(40.2%) | SCC: 70(32.0%) ADC: 91(41.6%) Others: 58(26.4%) | NA | NA | TILs+ | DFS | Surgery(100%) | ≥24 | 8 |
| Kilvaer  (2020) | Norway | Cohort Study | Full-text | 1990 to 2010 | 553(68%) | median: 67 | I: 232(42.0%) II: 185(33.5%) III: 136(24.5%) | SCC: 307(55.5%) ADC: 239(43.2%) Others: 7(1.3%) | CD3 CD4 CD8 CD20 CD45RO | TC  TS | CD>1000 cells/mm^2^ CD4>550 cells/mm^2^ CD8>500 cells/mm^2^ CD20>400 cells/mm^2^ CD45RO>250 cells/mm^2^ | OS | Surgery(100%) Surgery+PORT(30.4%) | NA | 7 |
| Kim(2019) | Korea | Cohort Study | Full-text | 2008 to 2012 | 146(47.3%) | median: 63.7  (range: 35-85) | I: 95(65.1%) II: 25(17.1%) III: 13(8.9%) IV: 13(8.9%) | ADC: 146(100%) | NA | TC  TS | TIL≥50% | PFS  OS | Surgery(100%) | median: 48 (range: 0-86) | 8 |
| Kikuchi  (2007) | Japan | Cohort Study | Full-text | 1982 to 1994 | 161(68.3%) | NA | I: 95(59.0%) NA: 66(41.0%) | SCC: 68(42.2%) ADC: 83(51.6%) Others: 10(6.2%) | CD8 | TC  TS | CD8>20% | OS | Surgery(100%) | median: 112 | 8 |
| Kinoshita  (2020) | Japan | Cohort Study | Full-text | 2003 to 2012 | 203(43.3%) | mean: 68 (range: 34-85) | IA: 203(100%) | ADC: 203(100%) | CD8 FOXP3 | NA | CD8+: ≥50 cells/0.04 mm^2^ FOXP3+: ≥20 cells/0.04 mm^2^ | OS  DFS | Surgery(100%) | NA | 8 |
| Koh(2017) | Korea | Cohort Study | Full-text | NA | 378(NA) | NA | NA | SCC: 378(100%) | CD103 | TC  TS | CD103+ | DFS | Surgery(100%) | NA | 6 |
| Kadota  (2015) | United States | Cohort Study | Full-text | 1999 to 2009 | 331(59.6%) | median: 72  (range: 39-88) | I: 190(57.4%) II: 93(28.1%) III: 48(14.5%) | SCC: 331(100%) | CD3 CD4 CD8 CD20 Foxp3 | TC | NA | OS | Surgery(82.5%) Surgery+ACT (17.5%) | NA | 6 |
| Kayser  (2012) | Germany | Cohort Study | Full-text | 1989 to 2007 | 232(72.0%) | median: 65  (range: 35-83) | I: 91(39.2%) II: 59(25.4%) III: 77(33.2%) IV: 3(1.3%) NA: 2(0.9%) | SCC: 93(40.1%) ADC: 75(32.3%) Others: 64(27.6%) | CD3 CD4 CD8 CD25 | TS | CD3+ CD4+ CD8+ CD25+ | OS | Surgery(100%) | mean: 32 (range: 1-210) | 7 |
| Kose  (2017） | Turkey | Cohort Study | Full-text | NA | 48(83.3%) | median: 62 (range: 43-78) | IB: 21(43.8%) II: 14(29.2%) III: 13(27.0%) | SCC: 24(50%) Non-SCC: 24(50%) | FOXP3 | TC | ≥25% | OS  DFS | Surgery(87.5%) Surgery+ACT (33.3%) | mean: 49 (range: 6-128) | 8 |
| Kuykendal(2015) | United States | Cohort Study | Abstract | 1996 to 2010 | 458(46%) | median: 67 | NA | SCC: 46(10%) ADC: 348(76%) Others: 64(14%) | CD3 | NA | NA | OS | Surgery(100%) | NA | 6 |
| Lee(2020) | United States | Cohort Study | Full-text | January 1  to December 30, 2009 | 120(43.3%) | median: 68.5 (range: 46-91) | I: 82(68.3%) II: 17(14.2%) III: 17(14.2%) IV: 4(3.3%) | ADC: 120(100%) | CD3 CD20 | TC  TS | CD3+ CD20+ | NA | Surgery(75%) Surgery+PORT(25%) | NA | 6 |
| Meng  (2018) | China | Cohort Study | Full-text | 2009 to 2011 | 197(65%) | NA | I: 112(56.9%) II: 45(22.8%) III: 40(20.3%) | SCC: 85(43.1%) Non-SCC: 112(56.9%) | CD4 CD8 FOXP3 | TC  TS | CD4>5% in TC,  ＞25% in TS, CD8>5% in TC,  ＞50% in TS FOXP3>2% in TC,＞20% in TS | OS | Surgery(49%) Surgery+PORT(51%) | NA | 7 |
| Mezquita  (2019) | France | Cohort Study | Abstract | 2009 to 2017 | 221(64%) | median: 63 | III/IV: 221(100%) | ADC: 162 (73%) Others: 59(27%) | NA | TS | TIL≥10% | PFS  OS | ICI or CT | NA | 5 |
| Mlika  (2022) | Tunisia | Cohort Study | Full-text | 2011 to 2015 | 39(74.4%) | mean: 59.6 | I/II: 7(18%) III/IV: 32(82%) | SCC: 9 (23%) ADC: 28(72%) Others: 2(5%) | CD3 CD8 FOXP3 | TC  TS | >0/HMF | OS  RFS  ReFS | Surgery: 25(64%) Surgery+NCT: 18(46%) | median: 32 (range: 1-73) | 8 |
| O’Callaghan(2015) | Australia | Cohort Study | Full-text | 2001 to 2005 | 196(58.7%) | mean: 65.5  (range: 41-86) | I: 103(52.6%) II: 44(22.5%) III: 39(19.9%)  NA:10(5.0%) | SCC: 85 (43.4%) ADC: 84(42.9%) Others: 27(13.7%) | CD3 CD8 FOXP3 | TC  TS | CD3>median CD8>median FOXP3>median | OS | Surgery(100%) | ≥60 | 7 |
| Ohtaki  (2018) | Japan | Cohort Study | Full-text | 2000 to 2016 | 95(86.3%) | median: 74  (range: 36-88) | I: 54(56.8%) II: 24 (25.3%) III: 14 (14.7%) IV: 3(3.2%) | LCNEC only: 77(81%) Combined LCNEC: 18(19%) | CD4 CD8 FOXP3 | TC  TS | CD4+ CD8+ FOXP3+ | OS | Surgery(100%) | median: 36.6 (range: 0.5-171) | 8 |
| Onion  (2017) | UK | Cohort Study | Full-text | 2010 to 2012. | 62(NA) | NA | I: NA II: NA III: NA | NA | CD3 CD8  FOXP3 | TC  TS | CD3+ CD8+  FOXP3+ | OS | Surgery(100%) | NA,followed  up to April 2015 | 6 |
| Petersen  (2006) | United States | Cohort Study | Full-text | 1996 to 2001 | 64(53.1%) | mean: 67 | I: 64(100%) | SCC: 22(34.3%) ADC: 30(46.9%) Others: 12(18.8%) | CD3 FOXP3 | TC | CD3+ FOXP3+ | OS | Surgery(100%) | ≥30 | 7 |
| Rakaee  (2018) | Norway | Cohort Study | Full-text | 1990 to 2010 | 537(68%) | NA | I: 226(42.1%) II: 181(33.7%) III: 130(24.2%) | SCC: 298(55.5%) ADC: 232(43.2%) Others: 7(1.3%) | NA | TC  TS | TIL>50% | OS/DSS/DFS | Surgery(100%) | median: 86 (range: 34–267) | 8 |
| Richardet  (2019) | Argentina | Cohort Study | Abstract | 2004 to 2014 | 187(72%) | NA | IIIB: NA IV: NA | SCC: 69(37%) ADC: 118(63%) | NA | NA | TILs+ | PFS | NA | NA | 6 |
| Ruffini  (2009) | Italy | Cohort Study | Full-text | 1993 to 2006 | 1290(84.0%) | mean: 64 | I: 714(55.3%) II: 265(20.5%) III: 214(12.6%)  NA:97(7.6%) | SCC: 549(42.6%) ADC: 495(38.4%) Others: 246(19.0%) | CD8 | TC | ≥20% | OS | Surgery(100%) | NA | 7 |
| Schulze  (2020) | Germany | Cohort Study | Full-text | 1998 to 2004 | 294(80%) | mean: 65.3 | I: 156(53.1%) II: 74 (25.2%) III: 64 (21.7%) | SCC: 141 (48%) ADC: 112 (38%) Others: 41(14%) | CD4 CD8 FOXP3 | TC  TS | CD4≥20% CD8≥20% FOXP3≥1% | PFS  OS | Surgery(70.1%) Surgery+NCT (29.9%) | median: 89.5 | 8 |
| Shimizu  (2010) | Japan | Cohort Study | Full-text | NA | 100(60%) | NA | I: 68(68.0%) II: 14 (14.0%) III: 18(18.0%) | SCC: 31 (31%) ADC: 69 (69%) | FOXP3 | TC | ≥10/HPF | RFS | Surgery(100%) | ≥24 | 7 |
| Suzuki  (2013) | United States | Cohort Study | Full-text | 1995 to 2009 | 956(62.1%) | median: 68.5 (range: 23-96) | I: 956(100%) | ADC: 956(100%) | FOXP3 | TS | Score≥2 | RFP | Surgery(100%) | NA | 6 |
| Shirasawa  (2022) | Japan | Cohort Study | Full-text | 2007 to 2020 | 551(75%) | median: 65 (range: 28-86) | II: 25(4.5%) III: 464(84.2%) NA: 62(11.3%) | SCC: 174(32%) NON-SCC: 377(68%) | CD8 | TC | ≥100/mm2 | PFS | CRT(79.5%) CRT+ICI(20.5%) | median: 25.7 (range: 2.7-150.7) | 8 |
| Soo(2014) | Australia | Cohort Study | Abstract | NA | 105(70%) | median: 64 | I: 62(59.0%)  NA:43(41.0%) | ADC: 60(57.1%)  Other: 45(42.9%) | CD3 CD8 FOXP3 | NA | ≥median | DSS | Surgery(100%) | NA | 6 |
| Souza  (2012) | Brazil | Cohort Study | Full-text | NA | 65(60%) | median: 62 (range: 34-82) | I: 20(30.8%) II: 33(50.8%) III: 12(18.4%) | SCC: 20(30.8%) ADC: 38(58.5%) Others: 7(10.7%) | CD3 CD4 CD8 | NA | CD3+ CD4+ CD8+ | Death rate | Surgery(100%) | median: 23 (range: 1–151) | 8 |
| Tan(2021) | China | Cohort Study | Full-text | 2013 to 2016 | 167(71.3%) | median: 61 (range: 35-84) | I: 48(28.8%) II: 42(25.1%) III: 77(46.1%) | SCC: 77(46.1%) ADC: 90(53.9%) | CD45RO | TC  TS | CD45RO+ | OS | Surgery(100%) | NA | 7 |
| Tao(2012) | Japan | Cohort Study | Full-text | 2001 to 2008 | 87(64.4%) | NA | NA | SCC: 19(21.8%) ADC: 61(70.1%) Others: 7(8.1%) | FOXP3 | TC | ≥20% | OS/RFS | Surgery(100%) | NA | 6 |
| Teng  (2016) | China | Cohort Study | Full-text | 2004 to 2012 | 126(67%) | median: 61 (range: 38-78) | I: 126(100%) | SCC: 42(33%) ADC: 57(45%) Others: 27(22%) | CD8 FOXP3 | TS | CD8+: ≥30% Foxp3: ≥45/HPF | DFS  OS | Surgery(100%) | median: 80 | 8 |
| Tian(2015) | China | Cohort Study | Full-text | 2004 to 2007 | 129(70.5%) | median: 61 (range: 32-83) | I: 48(37.2%) II: 29 (22.5%) III: 52 (40.3%) | SCC: 61 (47.3%) ADC: 48 (37.2%) Others: 20(15.5%) | CD3 CD8 | TC  TS | H‑score: 5-12 | OS | Surgery(100%) ACT: 92(71.32%) | median: 36.0  (range: 6-109) | 8 |
| Usó(2016) | Spain | Cohort Study | Full-text | 2004 to 2013 | 122(85.4%) | median: 65 (range: 26-85) | I: 72(59.0%) II: 26(21.3%) IIIA: 24(19.7%) | SCC: 58(47.5%) ADC: 51(41.8%) Others: 13(10.7%) | CD4 CD8 FOXP3 | TC  TS | CD4≥median CD8≥median FOXP3+ | NA | Surgery(100%) | median: 53.3 (range: 1-113) | 8 |
| Wakabayashi(2003) | Japan | Cohort Study | Full-text | 1976 to 1994 | 178(66.3%) | mean: 63.6 | I: 107(60.1%) II: 23(12.9%) III: 48(27.0%) | SCC: 83(46.6%) ADC: 95(53.4%) | CD4 CD8 | TC  TS | median | OS | Surgery(86.0%) | NA | 7 |
| Yang  (2018) | China | Cohort Study | Full-text | 2011 to 2015 | 178(63.5%) | median: 62 (range: 24-77) | I: 85(47.8%) II: 47(26.4%) III: 39(21.9%) IV: 7(3.9%) | SCC: 41(23.0%) ADC: 137(77.0%) | CD8 | TC | CD8≥5% | OS | Surgery(100%) | NA | 6 |
| Yazdi  (2016) | Netherlands | Cohort Study | Full-text | NA | 197(50.3%) | mean: 66 (range: 37-90) | I: 62(31.4%) II: 74(37.6%) III: 35(17.8%) IV: 26(13.2%) | ADC: 197(100%) | CD8 | TC  TS | CD8+ | OS | Surgery(100%) | ≤60 | 7 |

Overall survival (OS),Disease-free survival (DFS),Progression-free survival(PFS),Confidence Intervals (CIs),Hazard Ratios (HRs),Objective Response Rate(ORR),Disease Control Rate(DCR),Tumor Compartment(TC),Tumor Strom(TS),Radiation Treatment(RT),Recurrence-free survival(RFS),Disease-specific survival(DSS),Distant metastasis-free survival(DMFS),Recurrence-free survival(ReFS),Mean survival time(MST),First had tumour surgical resection followed by treatment with chemotherapy and radiotherapy(PORT),Chemotherapy and radiotherapy(DRC),Adjuvant chemotherapy(ACT),Chemotherapy(CT),Immune Checkpoint Inhibitor(ICI),Neoadjuvant chemotherapy(NCT),Chemoradiotherapy(CRT),Adenocarcinoma(ADC),Squamous Cell Carcinoma(SCC),Lung sarcomatoid carcinoma(LSC),Large-cell neuroendocrine carcinoma of the lung(LCNEC)
